# Tailored Fabrication of Carbon Dot Composites with Full‐Color Ultralong Room‐Temperature Phosphorescence for Multidimensional Encryption

**DOI:** 10.1002/advs.202103833

**Published:** 2021-11-19

**Authors:** Yuanfei Ding, Xueliang Wang, Miao Tang, Huibin Qiu

**Affiliations:** ^1^ School of Chemistry and Chemical Engineering Frontiers Science Center for Transformative Molecules State Key Laboratory of Metal Matrix Composites Shanghai Jiao Tong University Shanghai 200240 China

**Keywords:** boron, carbon dots, encryption, full‐color, room‐temperature phosphorescence

## Abstract

Ultralong room‐temperature phosphorescence (RTP) is highly useful for information encryption, organic electronics, bioelectronics, etc. However, the preparation of related metal‐free materials with multiple colors across the full spectrum remains a major challenge. Herein, a facile method is developed to fabricate boron‐doped carbon dot (B‐CD) composites with full‐color long lifetime RTP continuously tailorable in the range of 466–638 nm simply by pyrolysis of the citric acid and boric acid precursors with various mass ratios at different temperatures. This leads to the formation of luminescent B‐CD centers in a rigid polycrystalline B_2_O_3_ matrix, which effectively stabilizes the triplet excited states of B‐CDs. Thus, the composites become phosphorescent over a relatively long period (5–12 s) after the removal of the irradiation source. Meanwhile, the increased particle size and oxidation degree of B‐CDs obtained at larger citric acid feeding or higher pyrolysis temperature continuously shift the phosphorescence from blue to red. Due to the formation of multiple luminescence centers, the RTP can also be finely modulated by the excitation wavelength. The resulting B‐CD composites with highly tunable long lifetime RTP further allow a variety of distinctive applications in multidimensional encryption handily utilizing space, time, and color variations.

## Graphical abstract

Simple pyrolysis of citric acid and boric acid leads to the formation of carbon dot composites with highly tailorable full‐color ultralong room temperature phosphorescence, which subsequently favors a variety of distinctive applications in multidimensional encryption.

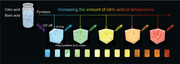

## 1 Introduction

Room‐temperature phosphorescence (RTP) with ultralong emission lifetime has been widely used in high‐sensitivity bioimaging,^[^ ^1^ ^]^ information anti‐counterfeiting,^[^ ^2^ ^]^ decoration,^[^ ^3^ ^]^ optoelectronic devices.^[^ ^4^ ^]^ In addition to the conventional metal‐containing complexes, purely organic phosphorescent materials are currently emerging as a highly promising supplement owing to their low cost and versatile fabrication pathways such as crystal engineering,^[^ ^5^ ^]^ host–guest doping,^[^ ^6^ ^]^ H‐aggregation.^[^ ^3^, ^4^, ^7^ ^]^ However, most of these materials demand relatively rigorous synthetic conditions and normally fail to widely adjust the RTP color in a single system under ambient conditions, which substantially limits their practical application.^[^ ^3^, ^7^, ^8^ ^]^

Recently, carbon dots (CDs) have triggered tremendous attention in constructing RTP materials because of their easy preparation, high photostability, and prominent biocompatibility.^[^ ^9^ ^]^ Normally, the phosphorescence of CDs can be generated and enhanced by heteroatom doping^[^ ^10^ ^]^ or embedding CDs into a host matrix such as polyvinyl alcohol, silica, inorganic salts, urea^[^ ^11^ ^]^ or immobilizing CDs on a substrate.^[^ ^12^ ^]^ For example, Hu and co‐workers found that boric acid (BA) can serve as a universal host to react with different CDs to acquire blue, green, green‐yellow, and orange RTP.^[^ ^11b^ ^]^ Ren et al. developed a time‐dependent RTP system by printing CDs on paper and found that the phosphorescence color changed from orange to green after irradiation by a specific light.^[^ ^12^ ^]^ Nevertheless, it remains a major difficulty to handily prepare CD materials with more colorful and especially longer wavelength (red) RTP emissions, in spite of a few recently attempts.^[^ ^11^, ^12^, ^13^ ^]^ Besides, the currently methods for RTP CD materials normally involve multiple preparation steps and precursors, which appears to be relatively tedious and less controllable in consideration of large‐scale fabrication.^[^ ^10^, ^11^ ^]^

Herein, we develop a facile and scalable approach to prepare CD composites with full‐color ultralong RTP simply by pyrolysis of a mixture of BA and citric acid (CA). The RTP emission continuously shifts from blue to red color upon increasing the CA feeding and the pyrolysis temperature, and appears to be excitation‐dependent. Fine analysis of the composites indicates that the RTP color is predominantly determined by the particle size and oxidation degree of CDs that embedded in the polycrystalline B_2_O_3_ matrix. We also explore the potential application of these RTP CD composites in multidimensional information encoding and anti‐counterfeiting.

## 2 Results and Discussion

### 2.1 Fabrication of Full‐Color Tunable RTP Materials

**Figure 1:**
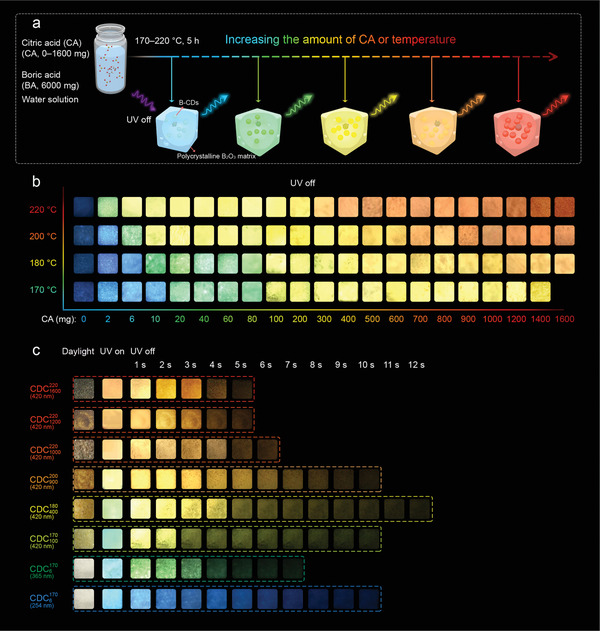
Fabrication of B‐CD composites. a) Schematic representation of the fabrication process of B‐CD composites with tunable full‐color ultralong RTP. b) Optical images of B‐CD composites prepared from CA and BA at different reaction conditions (*x* is the weight of CA in milligrams, the weight of BA is 6000 mg, the reaction temperature is listed on the left) after ceasing the excitation light under ambient conditions. c) Optical images of ${\mathrm{CDC}}_{6}^{170}$, ${\mathrm{CDC}}_{100}^{170}$, ${\mathrm{CDC}}_{400}^{180}$, ${\mathrm{CDC}}_{900}^{200}$, ${\mathrm{CDC}}_{1000}^{220}$, ${\mathrm{CDC}}_{1200}^{220}$, and ${\mathrm{CDC}}_{1600}^{220}$ under ambient sunlight, excited with UV light (wavelengths are denoted in the brackets), and at different delay times after switching off the excitation light.

**Figure 2:**
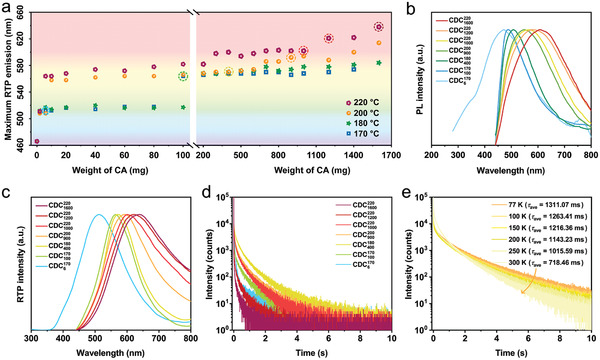
Optical properties of B‐CD composites. a) Maximum emission peaks of B‐CD composites prepared at different CA feeding and pyrolysis temperature. The optimal excitation wavelength of B‐CD composites changes from 260 to 420 nm when the amount of CA reaches 100 mg for 170 °C, 200 mg for 180 °C, 10 mg for 200 °C, and 6 mg for 220 °C, respectively. The circles correspond to ${\mathrm{CDC}}_{6}^{170}$, ${\mathrm{CDC}}_{100}^{170}$, ${\mathrm{CDC}}_{400}^{180}$, ${\mathrm{CDC}}_{900}^{200}$, ${\mathrm{CDC}}_{1000}^{220}$, ${\mathrm{CDC}}_{1200}^{220}$, and ${\mathrm{CDC}}_{1600}^{220}$. b) Normalized steady‐state photoluminescence spectra, c) normalized phosphorescence spectra, and d) time‐resolved phosphorescence decay curve of ${\mathrm{CDC}}_{6}^{170}$, ${\mathrm{CDC}}_{100}^{170}$, ${\mathrm{CDC}}_{400}^{180}$, ${\mathrm{CDC}}_{900}^{200}$, ${\mathrm{CDC}}_{1000}^{220}$, ${\mathrm{CDC}}_{1200}^{220}$, and ${\mathrm{CDC}}_{1600}^{220}$ under ambient conditions (excitation wavelength: 260 nm for ${\mathrm{CDC}}_{6}^{170}$, 420 nm for ${\mathrm{CDC}}_{100}^{170}$, ${\mathrm{CDC}}_{400}^{180}$, ${\mathrm{CDC}}_{900}^{200}$, ${\mathrm{CDC}}_{1000}^{220}$, ${\mathrm{CDC}}_{1200}^{220}$, and ${\mathrm{CDC}}_{1600}^{220}$). e) Time‐resolved phosphorescence decay curve of ${\mathrm{CDC}}_{400}^{180}$ measured from 77 to 300 K with excitation wavelength of 420 nm.

To fabricate the CD composites, CA and excess BA were first mixed by dissolving in hot water and then dried and melted at a higher temperature (170‐–220 °C) (**Figure** **1**a). Neither the CA nor the BA powder presented phosphorescence at room temperature, but the resulting composites exhibited bright long‐lived luminescence after the removal of irradiation (Figure 1b). The luminescence at ambient conditions can persist 5–12 s after ceasing the light irradiation (Figure 1c), indicating an ultralong RTP feature. As the amount of CA and the reaction temperature increased, the long‐lived luminescence color can be continuously customized from blue to red (Figures 1b and **2**a; Figure S1 and Table S1, Supporting Information). When the reaction temperature was 170 °C, the long‐lived luminescence color changed from blue (466 nm) at a low amount of CA to yellow (574 nm) at a high amount of CA. When the reaction temperature was increased to 180 and 200 °C, the long‐lived luminescence colors can be redshifted to orange (582 nm) and red (614 nm), respectively, at high amounts of CA. Moreover, when the reaction temperature was set at 220 °C, the long‐lived luminescence color became yellow (566 nm) at a low amount of CA and further shifted to red (638 nm) at a high CA feeding. It should be noted that the composite derived from pure BA exhibited weak blue persistent emission, but the emission was independent of the reaction temperature (Figures 1b and 2a).

### 2.2 Photophysical Properties

To verify the ultralong RTP characteristics and investigate the multicolor emission, seven typical composites with blue to red long‐lived luminescence emission were chosen as model samples for further analysis. According to the reaction condition, the samples were labeled as ${\mathrm{CDC}}_{6}^{170}$, ${\mathrm{CDC}}_{100}^{170}$, ${\mathrm{CDC}}_{400}^{180}$, ${\mathrm{CDC}}_{900}^{200}$, ${\mathrm{CDC}}_{1000}^{220}$, ${\mathrm{CDC}}_{1200}^{220}$, and ${\mathrm{CDC}}_{1600}^{220}$, respectively, where the subscript denotes the amount of CA (mg), and the superscript denotes the reaction temperature (°C). The luminescent properties of the composites were recorded under ambient conditions. Steady‐state photoluminescence (PL) spectra revealed main emission bands ranging from 418 to 614 nm (Figure 2b), which can be attributed to the fluorescence emission according to their nanosecond lifetime (Figure S2 and Table S2, Supporting Information). Upon switching off the optimal excitation light, the composites showed long‐lived emission with broad emission bands centered from 512 to 638 nm (Figure 2c). From the time‐resolved decay curve of the long‐lived luminescence (Figure 2d; Table S3, Supporting Information), the average lifetimes of the composites were calculated to be 113.90–581.76 ms, indicating an ultralong phosphorescence emission feature. With a decrease of the temperature, the average lifetimes of the composites apparently increased due to the suppression of nonradiative transitions and reached up to a maximum value (1.31 s for ${\mathrm{CDC}}_{400}^{180}$) at 77 K (Figure 2e; Figure S3 and Tables S4–S10, Supporting Information), reflecting a phosphorescence nature rather than delayed fluorescence.^[^ ^4^, ^10^, ^11^ ^]^ To quantitatively estimate the RTP emission, the absolute photoluminescence efficiencies (*Ф*_pl_) were determined to be 0.71–29.98%, derived from which, RTP efficiency (*Φ*_phos_) values of 0.42–13.74% were obtained (Table S11 and Figure S4, Supporting Information). Moreover, the PL and RTP of the composites can be excited by a wide range of excitation lights (Figures S5–S8, Supporting Information). The RTP emission of the composites showed excitation‐dependent behavior (Figure S7, Supporting Information), which can be attributed to the existence of multiple triplet‐excited states in the composites (Figure S9, Supporting Information) as confirmed by the tri‐exponential decay features (Figure 2d; Table S3, Supporting Information).^[^ ^13^, ^14^ ^]^ Compared with${\mathrm{CDC}}_{6}^{170}$, ${\mathrm{CDC}}_{100}^{170}$, and ${\mathrm{CDC}}_{400}^{180}$, the RTP emission of ${\mathrm{CDC}}_{900}^{200}$, ${\mathrm{CDC}}_{1000}^{220}$, ${\mathrm{CDC}}_{1200}^{220}$, and ${\mathrm{CDC}}_{1600}^{220}$ was less affected by the excitation wavelength (Figure S7, Supporting Information), which was probably due to the elimination of some luminescence centers at higher pyrolysis temperatures.

### 2.3 Mechanism Study of the Full‐Color Tunable RTP

**Figure 3:**
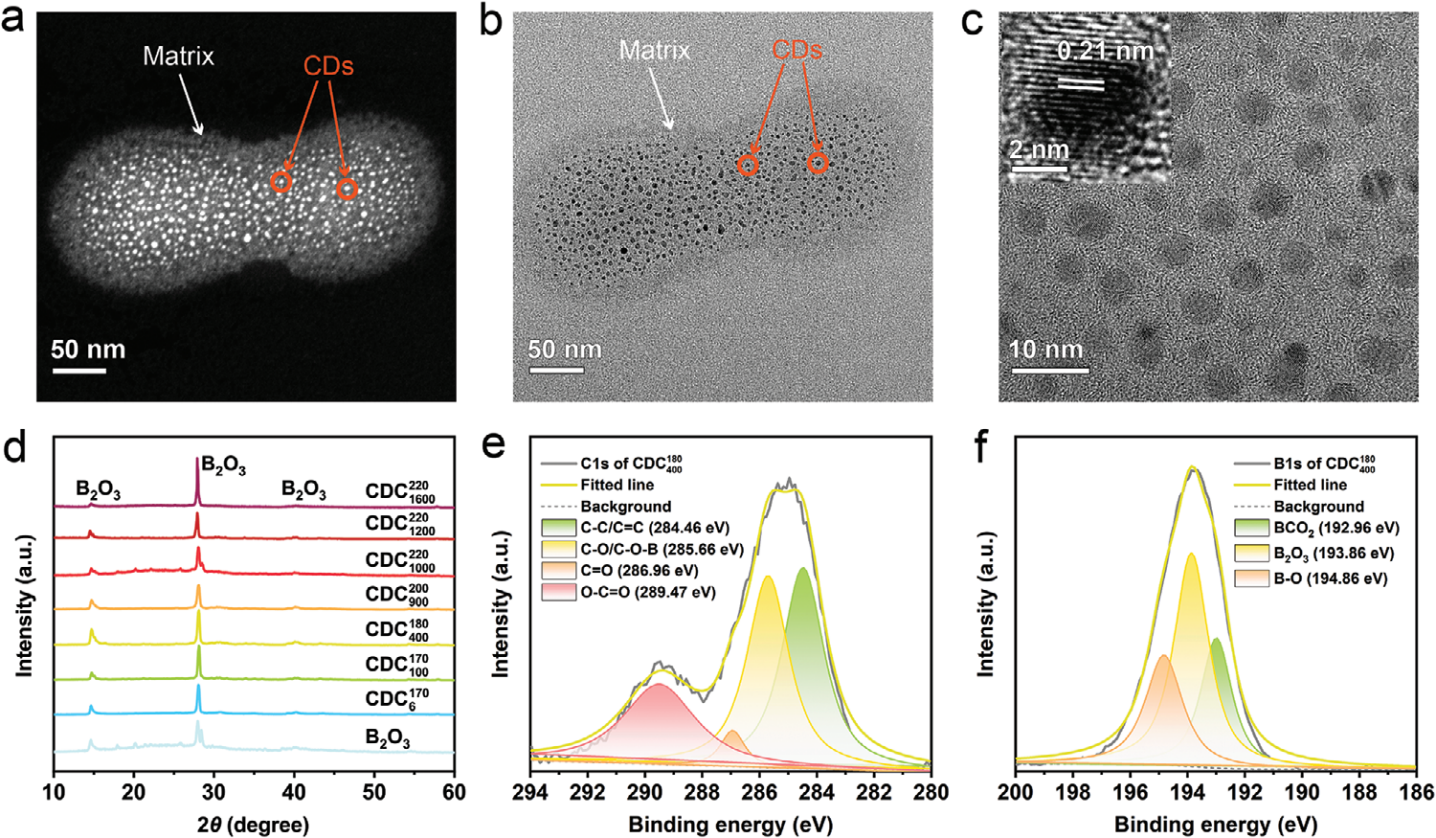
Structural analysis of B‐CD composites. a) HAADF‐STEM, b) TEM, and c) high‐resolution TEM images of ${\mathrm{CDC}}_{400}^{180}$ (Insets are the corresponding lattice fringes). d) XRD patterns of B‐CD composites. e) High‐resolution XPS C1s and f) B1s spectra of ${\mathrm{CDC}}_{400}^{180}$.

**Figure 4:**
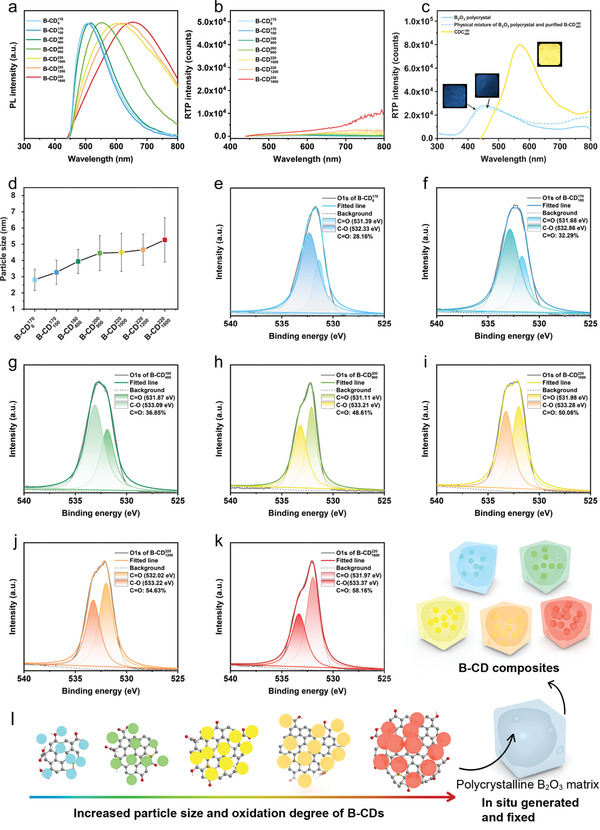
Origin of full‐color RTP. a) Steady‐state photoluminescence and b) phosphorescence spectra of B‐CDs purified from the B‐CD composites by treating with methanol. c) Phosphorescence spectra of B_2_O_3_ crystal, ${\mathrm{CDC}}_{400}^{180}$, and the physical mixture of purified ${B-\mathrm{CD}}_{400}^{180}$ and B_2_O_3_ crystal under ambient conditions (inset are the corresponding optical images). d) Particle size distribution of B‐CDs embedded in the polycrystalline B_2_O_3_ matrix. e–k) High‐resolution XPS O1s spectra of e) ${B-\mathrm{CD}}_{6}^{170}$, f) ${B-\mathrm{CD}}_{100}^{170}$, g) ${B-\mathrm{CD}}_{400}^{180}$, h) ${B-\mathrm{CD}}_{900}^{200}$, i) ${B-\mathrm{CD}}_{1000}^{220}$, j) ${B-\mathrm{CD}}_{1200}^{220}$, and k) ${B-\mathrm{CD}}_{1600}^{220}$ purified from the B‐CD composites. l) Schematic illustration for the origin of tunable full‐color ultralong RTP of B‐CD composites.

Detailed structural characterization of the composites was conducted to understand the origin of RTP. High‐angle annular dark‐field scanning transmission electron microscopy (HAADF‐STEM) and transmission electron microscopy (TEM) images demonstrated the formation of CDs that uniformly dispersed in a matrix (**Figure** **3**a–c; Figure S10, Supporting Information). The average particle size of CDs in the composites gradually increased with an increase of the CA feeding and pyrolysis temperature, namely 2.8 nm for ${\mathrm{CDC}}_{6}^{170}$, 3.3 nm for ${\mathrm{CDC}}_{100}^{170}$, 4.0 nm for ${\mathrm{CDC}}_{400}^{180}$, 4.4 nm for ${\mathrm{CDC}}_{900}^{200}$, 4.5 nm for ${\mathrm{CDC}}_{1000}^{220}$, 4.7 nm for ${\mathrm{CDC}}_{1200}^{220}$, and 5.3 nm for ${\mathrm{CDC}}_{1600}^{220}$ (**Figure** **4**d; Figure S10, Supporting Information). High‐resolution TEM (HRTEM) images showed that all these CDs possess a highly crystalline structure with a lattice fringe spacing of 0.21 nm (Inset of Figure 3c; Figure S10, Supporting Information) corresponding to the (100) plane of graphene.^[^ ^15^ ^]^ X‐ray diffraction (XRD) patterns revealed the distinct characteristic peaks of B_2_O_3_ (Figure 3d), demonstrating the presence of a polycrystalline B_2_O_3_ matrix.^[^ ^11b^ ^]^

Fourier transform‐infrared spectroscopy (FTIR) and X‐ray photoelectron spectroscopy (XPS) were further employed to investigate the chemical structure of the composites. FTIR spectra of the CD composites and boric oxide displayed similar absorption bands at 3216, 1455, and 1193 cm^–1^, which can be assigned to –OH and B–O stretching vibrations (Figure S11, Supporting Information), indicating the presence of large amounts of B_2_O_3_ in the composites.^[^ ^11b^ ^]^ XPS survey scan showed that all of the seven composites were composed of C, O, and B elements (Figures S12–S15, Supporting Information). The XPS C 1s spectra indicated the presence of C–C/C═C (284.46 eV), C–O/C–O–B (285.66 eV), C═O (286.96 eV), and O–C═O (289.47 eV) bonds (Figure 3e; Figure S13, Supporting Information). The XPS B 1s spectra revealed the presence of BCO_2_ (192.96 eV), B_2_O_3_ (193.86 eV), and B–O (194.86 eV) (Figure 3f; Figure S14, Supporting Information).^[^ ^11^, ^16^ ^]^ These results demonstrated the existence of boron oxide and the codoping of boron and oxygen in the CDs. It was postulated that the B‐doped CDs (B‐CDs) as luminescence centers were in situ generated and tightly embedded in the polycrystalline B_2_O_3_ matrix (Figure S16, Supporting Information). The highly rigid B_2_O_3_ polycrystalline network with 3D spatial restriction can effectively suppress the nonradiative transition and stabilize the triplet excited states of B‐CDs by restricting the molecular motion and isolating the quenchers in the ambient environment.^[^ ^10b^ ^]^ The significance of the polycrystalline B_2_O_3_ matrix for the generation of RTP was confirmed by the fact that the B‐CDs purified from the composites failed to emit RTP after irradiation (Figure 4b). However, the ultralong RTP can be subsequently recovered by melting the mixture of pure B‐CDs and BA, where the B‐CDs were re‐embedded in the polycrystalline B_2_O_3_ matrix (Figure S17, Supporting Information). Besides, the long lifetime RTP was found to be absent in the compounds fabricated by treating the mixture of BA and CA at relatively low temperatures (100 and 150 °C) (Figure S18, Supporting Information), as the BA molecules were unable to be converted into B_2_O_3_ (the melting point of BA is 171 °C). Moreover, when the B‐CD composites were dispersed with water and then freeze‐dried, the ultralong phosphorescence disappeared probably because that B_2_O_3_ was converted to BA (Figure S19, Supporting Information).^[^ ^11b^ ^]^ When the freeze‐dried products were melted again at 170–220 °C, where BA was converted to B_2_O_3_, the colorful ultralong RTP appeared again (Figure S20, Supporting Information). Notably, the physical mixture of B_2_O_3_ crystals and pure B‐CDs only exhibited very weak blue phosphorescence belonging to the B_2_O_3_ crystals (Figure 4c), indicating that the spatial restriction of the B‐CDs by the B_2_O_3_ polycrystalline network was essential for the long lifetime RTP.

While the presence of a polycrystalline B_2_O_3_ matrix turns on the RTP, it appears that the B‐CDs themselves as luminescence centers determine the RTP color. With the increase of CA feeding and reaction temperature, the PL emission of pure B‐CDs bathochromic‐ally shifted from blue to red, which was consistent with the change of the RTP color of the B‐CD composites (Figures 1b,2, 4). It was found that with the increase of CA feeding and reaction temperature: (i) the particle size of B‐CDs gradually increased from 2.8 to 5.3 nm (Figure 4d; Figure S10, Supporting Information); (ii) while all the pure B‐CDs revealed high‐quality graphitic structure (intensity ratio of crystalline G band to disordered D band: 1.20–1.77) (Figures S21 and S22, Supporting Information) and similar elemental composition (C, B, and O) and stretching vibration bands (–OH: 3384.46 cm^–1^, carboxylic C═O: 1716.34 cm^–1^, sp^2^ C═C: 1400.07 cm^–1^, C–O: 1274.72 cm^–1^ and B‐C: 1051.01 cm^–1^) (Figures S23–S25, Supporting Information, also confirming the doping of boron), their oxidation degree (the relative amount of C═O) gradually increased from 28.16% to 58.16% (Figure 4e–k; Table S12, Supporting Information). Thus, the products formed at higher CA feeding and reaction temperature exhibited longer wavelength PL^[^ ^15^, ^17^ ^]^ and subsequently rendered redshifted RTP (Figure 4l).

### 2.4 Practicability of Full‐Color RTP Tunable B‐CD Composites

**Figure 5:**
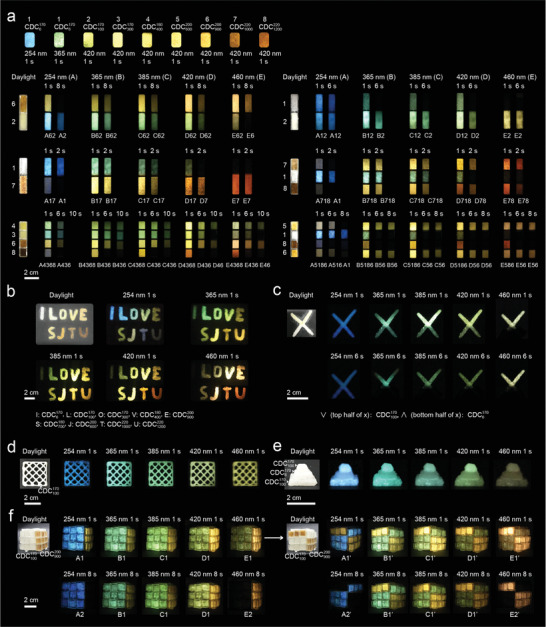
Information encoding, anti‐counterfeiting, and 3D encryption by B‐CD composites. a) Ultralarge capacity information encoding in barcodes fabricated by B‐CD composites. b) Light‐sensitive alphabetic security code derived from B‐CD composites. c) Light‐sensitive time‐resolved anti‐counterfeiting by B‐CD composites. d–f) Information encryption by 3D printing a gelatinous mixture of B‐CD composites and PDMS into d) lattice, e) pyramid structure, and f) Rubik's cube. All the luminescence images were taken after ceasing the excitation light.

On account of the full color and long afterglow to naked eyes, along with the light‐stimulated response properties, the B‐CD composites with gram scale fabrication capacity (Figure S26, Supporting Information) were used as information carriers for ultralarge capacity encoding. Eight composites (${\mathrm{CDC}}_{6}^{170}$, ${\mathrm{CDC}}_{100}^{170}$, ${\mathrm{CDC}}_{300}^{170}$, ${\mathrm{CDC}}_{400}^{180}$, ${\mathrm{CDC}}_{600}^{200}$, ${\mathrm{CDC}}_{900}^{200}$, ${\mathrm{CDC}}_{1000}^{220}$, and ${\mathrm{CDC}}_{1200}^{220}$) with different RTP colors were selected as the stripes of barcodes (**Figure** **5**a). The barcodes were fabricated by encapsulating the B‐CD composites (powder) in a desired poly(dimethylsiloxane) (PDMS) mold. The RTP colors of the barcodes were subsequently modulated by permutation of the B‐CD composites as well utilization of various excitation wavelengths. For information encoding, ${\mathrm{CDC}}_{6}^{170}$, ${\mathrm{CDC}}_{100}^{170}$, ${\mathrm{CDC}}_{300}^{170}$, ${\mathrm{CDC}}_{400}^{180}$, ${\mathrm{CDC}}_{600}^{200}$, ${\mathrm{CDC}}_{900}^{200}$, ${\mathrm{CDC}}_{1000}^{220}$, and ${\mathrm{CDC}}_{1200}^{220}$ were encoded as 1, 2, 3, 4, 5, 6, 7, and 8, respectively, and the exciting lights with wavelength of 254, 365, 385, 420, and 460 nm were encoded as A, B, C, D, and E, respectively. Taking the barcodes with a permutation of 62, 12, 17, 718, 4368, and 5186 for example, once ceasing the 254 nm light irradiation, these barcodes exhibited desired information (A62, A12, A17, A718, A4368, and A5186, respectively). However, such encoded information changed over time as a consequence of the different afterglow lifetimes of various B‐CD composites. In detail, both A17 and A718 became A1 after 2 s, while A12 can still be observed after 6 s, and A2 derived from A62 can be observed after 8 s. More abundantly, A5186 became A516 after 6 s and subsequently changed to A1 after 8 s. Moreover, A436 derived from A4368 was observed after 6 s and then disappeared after 10 s. Furthermore, when the excitation light was transformed to 365, 385, 420, and 460 nm, the same barcode also exhibited different encoded information due to the change of RTP color and afterglow lifetime of the composites upon excitation by different lights. The encoding capacity can be further enhanced by arranging multiple barcodes into an ordered or random array (Figure S27, Supporting Information). Such high‐level ultralarge capacity encoding apparently impedes mimicking or forging and thus would favor advanced practical applications in color coding of pharmaceutical packaging, paper currency, credit cards, and artwork.^[^ ^18^ ^]^

The B‐CD composites were further employed as an ideal material for anti‐counterfeiting. We first simply designed an alphabetic security code “I_LOVE_SJTU” using ${\mathrm{CDC}}_{6}^{170}$, ${\mathrm{CDC}}_{100}^{170}$, ${\mathrm{CDC}}_{300}^{170}$, ${\mathrm{CDC}}_{400}^{180}$, ${\mathrm{CDC}}_{900}^{200}$, ${\mathrm{CDC}}_{700}^{180}$, ${\mathrm{CDC}}_{600}^{200}$, ${\mathrm{CDC}}_{1000}^{220}$, and ${\mathrm{CDC}}_{1200}^{220}$ as the precursors (Figure 5b). Once stopping the 254 nm light irradiation, a colorful RTP pattern of “I_LOVE_SJTU” appeared. Once changing the excitation wavelength to 460 nm, only “LOVE_SJTU” can be observed while the character “I” disappeared since ${\mathrm{CDC}}_{6}^{170}$ barely showed any afterglow upon irradiation at 460 nm. Moreover, the character color of “I_LOVE_SJTU” changed when switching the excitation light to 365, 385, and 420 nm. Next, we prepared a cross “X” symbol with the top and bottom regions constituted by ${\mathrm{CDC}}_{6}^{170}$ and ${\mathrm{CDC}}_{100}^{170}$, respectively (Figure 5c). After irradiation by a 254 nm light, the pattern of “X” with blue color appeared and it retained for 6 s. When the excitation light was changed to 365, 385, and 420 nm, the pattern of “X” with green or olivine color appeared after ceasing the irradiation. However, only “√” can be observed after 6 s as a consequence of the short phosphorescence emission time of ${\mathrm{CDC}}_{6}^{170}$ under these excitation lights. Further, when using a 460 nm excitation light, only “√” with pale yellow color can be observed over the whole period after ceasing the irradiation majorly because that ${\mathrm{CDC}}_{6}^{170}$ is RTP silent upon irradiation by a longer wavelength light. These results indicated a great promise of the as‐fabricated B‐CD composites with full‐color ultralong lifetime for applications in the field of time‐resolved encryption.

To achieve more effective multistage data security, the encryption platform was expanded to 3D architectures. First, 3D lattice and pyramid structures were fabricated by printing a gelatinous mixture of B‐CD composites (${\mathrm{CDC}}_{100}^{170}$ for 3D lattice; ${\mathrm{CDC}}_{6}^{170}$ and ${\mathrm{CDC}}_{100}^{170}$ for pyramid) and PDMS (Figure 5d,e). Once stopping the excitation light, a bright 3D pattern appeared. When switching the excitation light from 254 to 365, 385, 420, and 460 nm, 3D images with colorful luminescence can be captured after stopping the irradiation, indicating a potential application of B‐CD composites in 3D display. Moreover, ring and braided fabric were also fabricated by the gelatinous mixture of B‐CD composites (${\mathrm{CDC}}_{100}^{170}$ for ring; ${\mathrm{CDC}}_{6}^{170}$, ${\mathrm{CDC}}_{100}^{170}$, and ${\mathrm{CDC}}_{400}^{180}$ for braided fabric) and PDMS (Figure S28, Supporting Information), and the objects showed bright RTP with a jade‐like feature. Next, we chose ${\mathrm{CDC}}_{100}^{170}$ and ${\mathrm{CDC}}_{900}^{200}$ as the information carriers and fixed them onto a Rubik's cube packed by 27 PDMS cubes (Figure 5f). Once stopping the 254 nm light irradiation, the Rubik's cube exhibited information A1. However, encrypted information A2 was observed after 8 s because of the longer afterglow lifetime of ${\mathrm{CDC}}_{100}^{170}$ under 254 nm light. Once changing the excitation wavelength to 460 nm, encrypted information E1 was observed, and then changed to E2 after 8 s owing to the longer afterglow lifetime of ${\mathrm{CDC}}_{900}^{200}$ under 460 nm light. Moreover, when switching the excitation light to 365, 385, and 420 nm, the encrypted information of B1, C1, and D1 can be observed over the whole period as a result of the similar afterglow lifetime of ${\mathrm{CDC}}_{100}^{170}$ and ${\mathrm{CDC}}_{900}^{200}$ under these excitation lights. When rotating the Rubik's cube, the encrypted information can be further reprogrammed, demonstrating higher level encryption.

## 3 Conclusion

In summary, we have developed a scalable method to fabricate B‐CD composites with long lifetime RTP highly tailorable in the range of 466–638 nm simply by controlled thermal pyrolysis of citric acid and boric acid. The 3D spatial restriction by a highly rigid polycrystalline B_2_O_3_ matrix was responsible for the long‐lifetime RTP. Meanwhile, the feature of the luminescent B‐CD centers ultimately determined the color of RTP of the composites. The full‐color RTP B‐CD composites showed prominent potential application in ultralarge capacity encoding, anti‐counterfeiting, and encryption in 3D architectures with multidimensional response to space, time, and color variations. In principle, the strategy developed in this work would be applicable to a rich array of combinations of small molecules for the preparation of CD composites with RTP activity and additional features (e.g., chiral, thermo‐responsive, humidity‐responsive). We are also improving the RTP stability of the CD composites in solution for more precise and diverse construction of complicated RTP architectures and devices.

## 4 Experimental Section

### Materials

Unless other noted, all reagents used in the experiments were purchased from commercial sources without further purification.

### Synthesis of Boron‐Doping Carbon Dot (B‐CD) Composites

6000 mg of boric acid (BA) and *x* mg (*x* = 0, 2, 6, 10, 20, 40, 60, 80, 100, 200, 300, 400, 500, 600, 700, 800, 900, 1000, 1200, 1600) of citric acid (CA) were firstly dissolved in 25 mL of deionized water in a 30 mL glass bottle. The mixture was heated at 110 °C overnight until it was fully dried. Afterward, the mixture was heated at 170, 180, 200, or 220 °C for 5 h and cooled down to room temperature naturally. A monolith can be obtained at the end and it was ground into powder for further study.

### Purification of B‐CDs from the Composites

Typically, 3720 mg of the B‐CD composite powder was dispersed in excess methanol in a glass bottle. The mixture was heated at 50 °C for 2 d to allow complete removal of the resulting trimethyl borate formed by B_2_O_3_ and methanol. Finally, pure B‐CDs can be obtained as a fine powder.

### Characterizations

HAADF‐STEM, TEM, and HRTEM images were collected in a Talos F200X G2 electron microscopy at an accelerating voltage of 200 kV. Samples were prepared by placing one drop of CD composite solution on an ultrathin carbon film‐coated copper grid, touching the edge of the droplet with a filter paper to remove excess liquid and allowing the grid to dry. Fourier transform infrared (FT‐IR) spectra were measured on a Nicolet 6700 FT‐IR spectrophotometer by the potassium bromide pellet technique. XRD patterns were obtained on an X‐ray diffractometer using Cu K*α* radiation (Bruker D8 Advance) by placing the powder sample on a non‐diffraction silicon wafer. XPS spectra of powder samples were obtained by an X‐ray photoelectron spectroscope (AXIS UltraDLD). Raman spectra were measured using a laser confocal micro‐raman spectroscope (Renishaw inVia Qontor) with an excitation wavelength of 532 nm by pressing the powder sample on a glass slide. UV–vis absorption spectra were taken on a Lambda 750S spectrophotometer. Samples were prepared by pressing the powder sample on dry barium sulfate. Steady‐state fluorescence and fluorescence lifetime spectra were obtained on an Edinburgh FLS1000 spectrophotometer equipped with a xenon arc lamp (Xe 900). Phosphorescence, phosphorescence excitation, and phosphorescence lifetime spectra were measured using an Edinburgh FLS1000 spectrophotometer equipped with a microsecond flash‐lamp (µF900). The powder samples were immobilized in the groove of a quartz plate before measurement. The absolute photoluminescence quantum yields (*Φ*_pl_) of all powder samples were measured using an Edinburgh FLS1000 spectrophotometer equipped with an integrating sphere.

## Conflict of Interest

The authors declare no conflict of interest.

## Supporting information

Supporting Information

## Data Availability

The data that supports the findings of this study are available in the supplementary material of this article.
