# Supplementary material for: Tailored Fabrication of Carbon Dot Composites with Full‐Color Ultralong Room‐Temperature Phosphorescence for Multidimensional Encryption
Source: Adv Sci (Weinh). 2021 Nov 19;9(3):2103833. doi: 10.1002/advs.202103833 (PMC8787396; doi:10.1002/advs.202103833)
Supplement: Supplementary file 1 — Supporting Information [file ADVS-9-2103833-s001.pdf]

ADVANCED SCIENCE
Open Access

Supporting Information

for *Adv. Sci.*, DOI: 10.1002/advs.202103833

Tailored fabrication of carbon dot composites with full-color ultralong room-temperature phosphorescence for multidimensional encryption

*Yuanfei Ding, Xueliang Wang, Miao Tang, and Huibin Qiu**

Supporting Information

Tailored fabrication of carbon dot composites with full-color ultralong room-temperature phosphorescence for multidimensional encryption

*Yuanfei Ding, Xueliang Wang, Miao Tang, and Huibin Qiu**

Table S1. Excitation wavelength (nm) corresponding to the optical images of B-CD composites shown in Figure 1b.

220 °C	254	365	420	420	420	420	420	420	420	420	460	460	460	460	460	460	460	460	460	460	460
200 °C	254	254	365	420	420	420	420	420	420	420	420	420	420	420	460	460	460	460	460	460	460
180 °C	254	254	254	365	365	365	365	365	420	420	420	420	420	420	420	420	420	420	420	420	420
170 °C	254	254	254	254	365	365	365	365	420	420	420	420	420	420	420	420	420	420	420	420	
x (mg) =	0	2	6	10	20	40	60	80	100	200	300	400	500	600	700	800	900	1000	1200	1400	1600

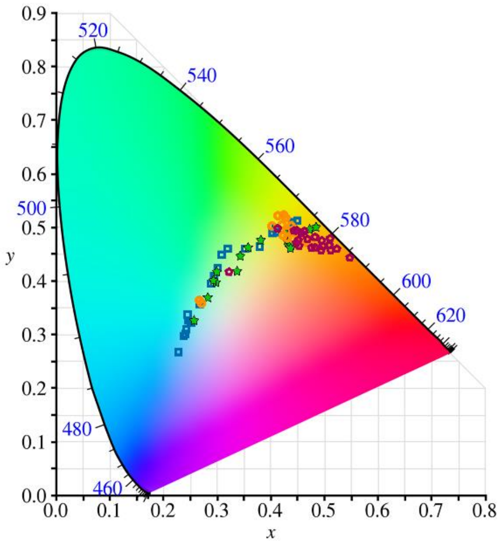

Figure S1. Chromaticity coordinates (x, y) calculated from RTP emission spectra of B-CD composites prepared from CA and BA at different reaction conditions.

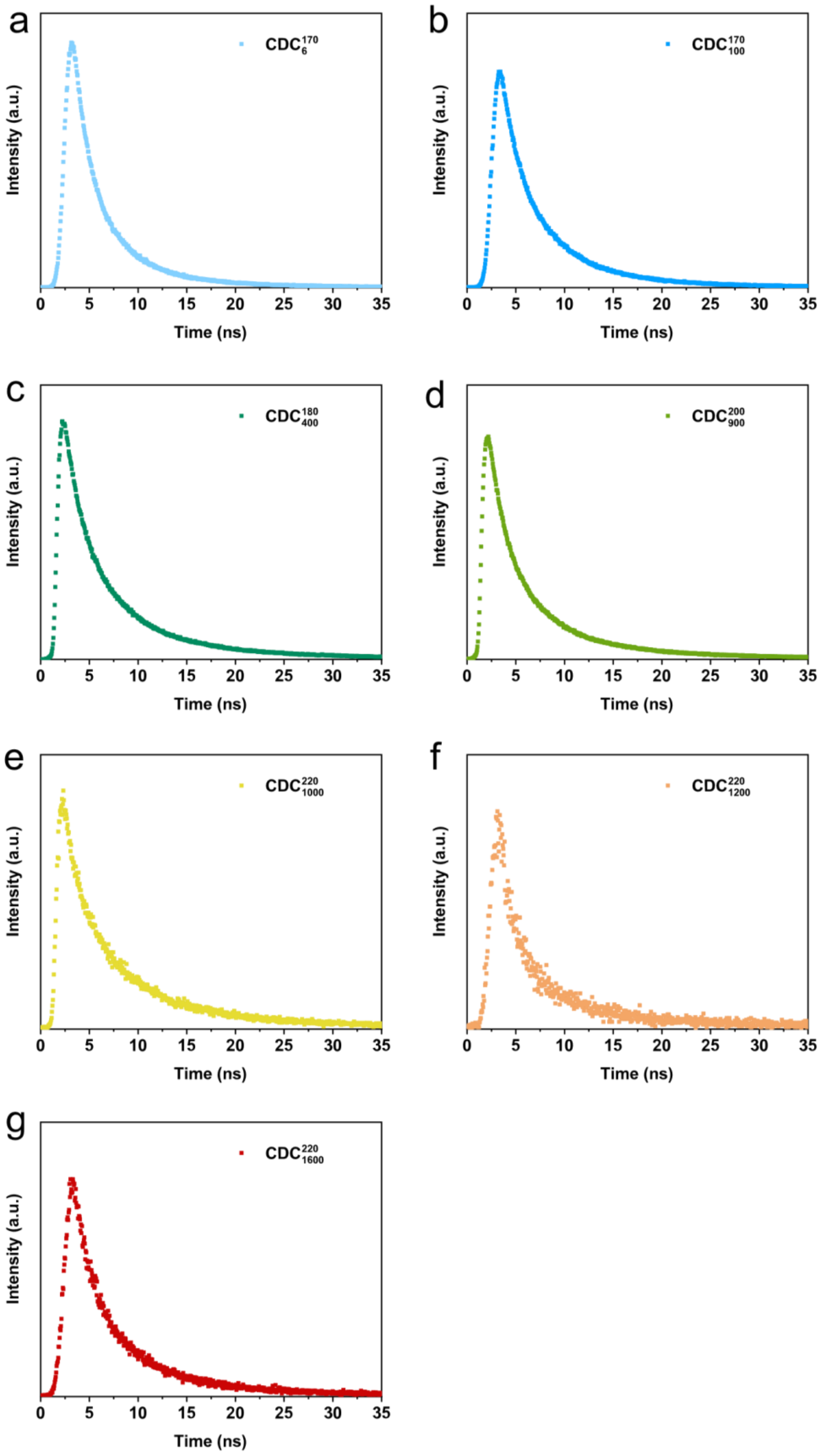

Figure S2. Time-resolved fluorescence decay curve of a) CDC_6^{170}, b) CDC_{100}^{170}, c) CDC_{400}^{180}, d) CDC_{900}^{200}, e) CDC_{1000}^{220}, f) CDC_{1200}^{220}, and g) CDC_{1600}^{220} monitored at 418, 452, 493, 556, 566, 586, and 614 nm, respectively, under ambient conditions.

Table S2. Fluorescence lifetimes of CDC_{6}^{170}, CDC_{100}^{170}, CDC_{400}^{180}, CDC_{900}^{200}, CDC_{1000}^{220}, CDC_{1200}^{220}, and CDC_{1600}^{220} under ambient conditions.

Sample	Excitation wavelength (nm)	Emission wavelength (nm)	τ_1 (ns)	B_1	τ_2 (ns)	B_2	τ_3 (ns)	B_3	τ_{ave} (ns)	χ^2
CDC_{6}^{170}	363	418	1.45	5262.79	3.86	5956.18	10.71	735.27	4.78	1.16
CDC_{100}^{170}	363	452	1.81	4349.27	4.89	5996.76	12.31	617.66	5.60	1.01
CDC_{400}^{180}	405	493	1.80	5652.58	5.16	8214.47	15.03	1357.54	7.45	1.05
CDC_{900}^{200}	460	556	1.59	6524.83	4.47	8780.69	12.49	1977.10	6.73	1.06
CDC_{1000}^{220}	405	566	2.87	782.62	9.83	406.92			7.33	1.05
CDC_{1200}^{220}	363	586	1.73	142.46	7.39	68.97			5.54	1.18
CDC_{1600}^{220}	363	614	2.78	615.01	9.19	234.25			6.36	1.16

Table S3. Phosphorescence lifetimes of CDC_{6}^{170} , CDC_{100}^{170} , CDC_{400}^{180} , CDC_{900}^{200} , CDC_{1000}^{220} , CDC_{1200}^{220} , and CDC_{1600}^{220} under ambient conditions.

Sample	Excitation wavelength (nm)	Emission wavelength (nm)	τ_1 (ms)	B_1	τ_2 (ms)	B_2	τ_3 (ms)	B_3	τ_{ave} (ms)	χ^2
CDC_{6}^{170}	260	512	36.60	1908.43	82.60	1155.73	965.98	54.13	280.09	1.25
CDC_{100}^{170}	420	564	18.36	1503.74	164.90	754.89	724.20	256.07	460.21	1.29
CDC_{400}^{180}	420	570	88.69	2488.76	357.93	2248.71	1172.80	416.91	581.76	1.10
CDC_{900}^{200}	420	592	49.84	2311.07	244.59	1484.68	881.26	384.92	481.32	1.15
CDC_{1000}^{220}	420	602	48.82	1546.70	217.00	1119.75	764.60	381.46	457.95	1.10
CDC_{1200}^{220}	420	621	6.02	2882.08	58.06	723.60	393.16	136.13	208.93	1.17
CDC_{1600}^{220}	420	638	3.97	1540.06	30.58	380.85	207.47	84.61	113.90	1.23

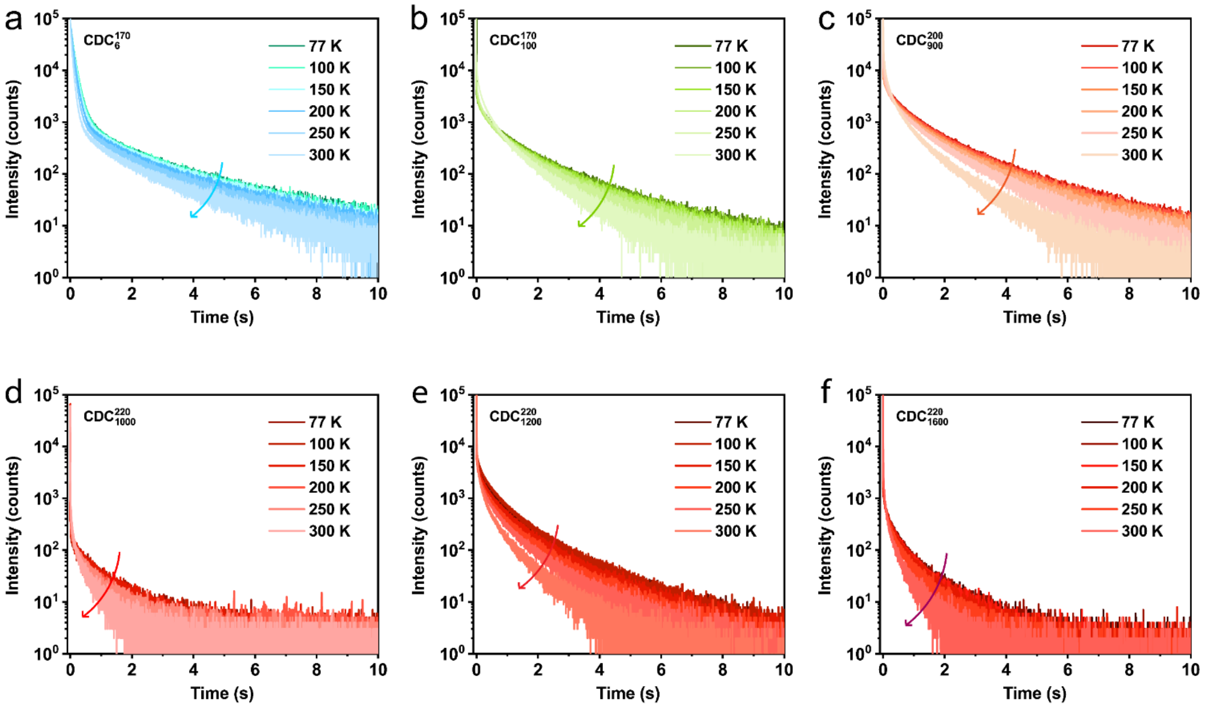

Figure S3. Time-resolved phosphorescence decay curve of a) CDC_6^{170}, b) CDC_{100}^{170}, c) CDC_{900}^{200}, d) CDC_{1000}^{220}, e) CDC_{1200}^{220}, and f) CDC_{1600}^{220} measured at temperatures from 77 to 300 K under optimal excitation wavelength.

Table S4. Phosphorescence lifetimes of CDC_6^{170} measured at temperatures from 77 to 300 K with excitation wavelength of 260 nm.

Temperature (K)	τ_1 (ms)	B_1	τ_2 (ms)	B_2	τ_3 (ms)	B_3	τ_{ave} (ms)	χ^2
77	101.12	44408.39	325.20	3502.15	1945.38	696.60	495.48	1.25
100	101.27	44118.67	318.81	3543.50	1936.26	698.17	493.58	1.19
150	95.17	43891.23	319.74	2984.44	1892.85	679.15	488.70	1.25
200	81.13	51874.20	271.79	3271.61	1792.53	660.54	430.70	1.29
250	65.84	55323.38	202.66	4316.85	1583.13	673.13	376.89	1.39
300	49.58	36144.63	139.34	5023.16	1244.26	657.98	363.99	1.29

Table S5. Phosphorescence lifetimes of CDC_{100}^{170} measured at temperatures from 77 to 300 K with excitation wavelength of 420 nm.

Temperature (K)	τ_1 (ms)	B_1	τ_2 (ms)	B_2	τ_3 (ms)	B_3	τ_{ave} (ms)	χ^2
77	109.34	1069.76	576.09	1271.79	1807.18	469.50	1159.06	1.15
100	81.00	1182.77	495.48	1369.93	1639.88	601.40	1113.91	1.11
150	51.54	1616.92	446.78	1422.19	1590.32	596.10	1077.43	1.18
200	45.50	2018.10	403.67	1482.54	1455.10	641.37	987.81	1.25
250	47.86	2943.07	379.72	1799.06	1380.70	629.34	865.87	1.24
300	51.75	5395.90	297.58	3329.02	1073.31	776.20	572.24	1.26

Table S6. Phosphorescence lifetimes of CDC_{400}^{180} measured at temperatures from 77 to 300 K with excitation wavelength of 420 nm.

Temperature (K)	τ_1 (ms)	B_1	τ_2 (ms)	B_2	τ_3 (ms)	B_3	τ_{ave} (ms)	χ^2
77	66.61	4238.57	542.83	4317.29	1857.50	2119.97	1311.07	1.20
100	97.97	2826.35	609.17	3638.61	1826.09	1725.41	1263.41	1.13
150	119.69	2390.37	634.64	3242.72	1810.86	1403.46	1216.36	1.11
200	82.53	3601.68	560.58	3750.05	1692.55	1656.47	1143.23	1.15
250	72.42	5869.83	504.73	4655.15	1562.88	1872.08	1015.59	1.26
300	97.71	11181.06	440.25	8764.01	1337.89	2113.68	718.46	1.29

Table S7. Phosphorescence lifetimes of CDC_{900}^{200} measured at temperatures from 77 to 300 K with excitation wavelength of 420 nm.

Temperature (K)	τ_1 (ms)	B_1	τ_2 (ms)	B_2	τ_3 (ms)	B_3	τ_{ave} (ms)	χ^2
77	124.01	2292.30	589.54	3050.65	1803.04	1182.37	1171.96	1.09
100	104.39	2536.61	555.83	3101.08	1749.42	1196.24	1138.58	1.12
150	104.86	2558.49	561.52	2900.34	1735.56	1075.35	1111.22	1.15
200	83.72	3192.05	507.63	2903.36	1619.96	1088.29	1034.94	1.20
250	97.66	3132.91	500.17	2760.18	1507.74	973.04	929.85	1.16
300	86.41	4487.99	348.97	3687.50	1085.52	955.69	593.17	1.15

Table S8. Phosphorescence lifetimes of CDC^{220}_{1000} measured at temperatures from 77 to 300 K with excitation wavelength of 420 nm.

Temperature (K)	τ_1 (ms)	B_1	τ_2 (ms)	B_2	τ_3 (ms)	B_3	τ_{ave} (ms)	χ^2
77	45.12	60.40	325.30	90.80	1059.41	58.77	800.77	1.00
100	68.84	53.13	309.88	73.82	999.83	60.45	779.20	1.03
150	53.58	63.55	292.53	88.20	981.59	67.90	763.15	1.11
200	20.06	74.96	196.27	101.05	888.66	73.25	714.68	1.20
250	14.18	39.11	153.41	97.90	723.76	73.07	592.88	1.18
300	38.93	140.98	149.41	133.10	575.47	62.38	389.13	1.11

Table S9. Phosphorescence lifetimes of CDC_{1200}^{220} measured at temperatures from 77 to 300 K with excitation wavelength of 420 nm.

Temperature (K)	τ_1 (ms)	B_1	τ_2 (ms)	B_2	τ_3 (ms)	B_3	τ_{ave} (ms)	χ^2
77	63.14	2911.16	351.10	2819.78	1175.28	1046.42	750.77	1.15
100	57.89	3280.40	335.17	3086.90	1132.54	1255.65	743.72	1.11
150	56.83	2383.61	321.90	2275.09	1091.47	954.92	723.03	1.10
200	52.27	2359.82	304.45	1923.75	1048.35	759.48	677.28	1.11
250	32.37	3449.50	207.63	2097.29	824.11	875.20	542.76	1.14
300	28.23	3490.69	129.62	2384.45	523.39	964.35	336.52	1.16

Table S10. Phosphorescence lifetimes of CDC_{1600}^{220} measured at temperatures from 77 to 300 K with excitation wavelength of 420 nm.

Temperature (K)	τ_1 (ms)	B_1	τ_2 (ms)	B_2	τ_3 (ms)	B_3	τ_{ave} (ms)	χ^2
77	36.81	609.65	210.31	528.35	782.85	207.42	511.30	1.13
100	20.92	1003.77	181.48	632.59	745.44	241.56	492.30	1.18
150	32.25	643.23	190.47	540.20	726.15	224.03	483.37	1.15
200	29.18	845.34	177.90	576.02	690.17	214.75	440.31	1.15
250	24.56	1141.29	135.91	673.73	574.70	234.21	355.97	1.16
300	44.03	581.42	163.15	385.08	509.76	123.29	287.00	1.12

Table S11. Photophysical parameters of CDC^{170}_{6} , CDC^{170}_{100} , CDC^{180}_{400} , CDC^{200}_{900} , CDC^{220}_{1000} , CDC^{220}_{1200} , and CDC^{220}_{1600} under ambient conditions.

Sample	CDC^{170}_{6}	CDC^{170}_{100}	CDC^{180}_{400}	CDC^{200}_{900}	CDC^{220}_{1000}	CDC^{220}_{1200}	CDC^{220}_{1600}
Excitation wavelength (nm)	260	420	420	420	420	420	420
Φ_{pl} (%)	5.76	29.98	20.92	6.26	3.81	1.71	0.71
Φ_{phos} (%)	3.39	13.74	10.44	4.84	2.00	0.97	0.42
Φ_{fluo} (%)	2.37	16.24	10.48	1.42	1.81	0.74	0.29

The absolute photoluminescence quantum yields (Φ_{pl}) of all solid-state composites were measured using an Edinburgh FLS1000 spectrophotometer equipped with an integrating sphere under ambient conditions. The phosphorescent (Φ_{phos}) and fluorescent (Φ_{fluo}) quantum yields of the composites were obtained from the following equation:

$$\Phi_{phos} = \frac{B}{A} \times \Phi_{pl}$$

$$\Phi_{fluo} = \frac{C}{A} \times \Phi_{pl}$$

A = B+C

where A, B and C represent the integral areas of total photoluminescence, phosphorescence and fluorescent spectra, respectively. The phosphorescence was separated from photoluminescence in a time-gated scheme (Figure S4).[1]

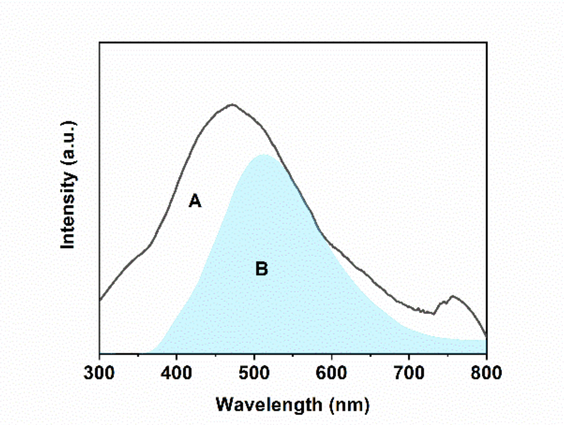

Figure S4. Schematic diagram of the integral area of photoluminescence and phosphorescence for quantum yield calculation.

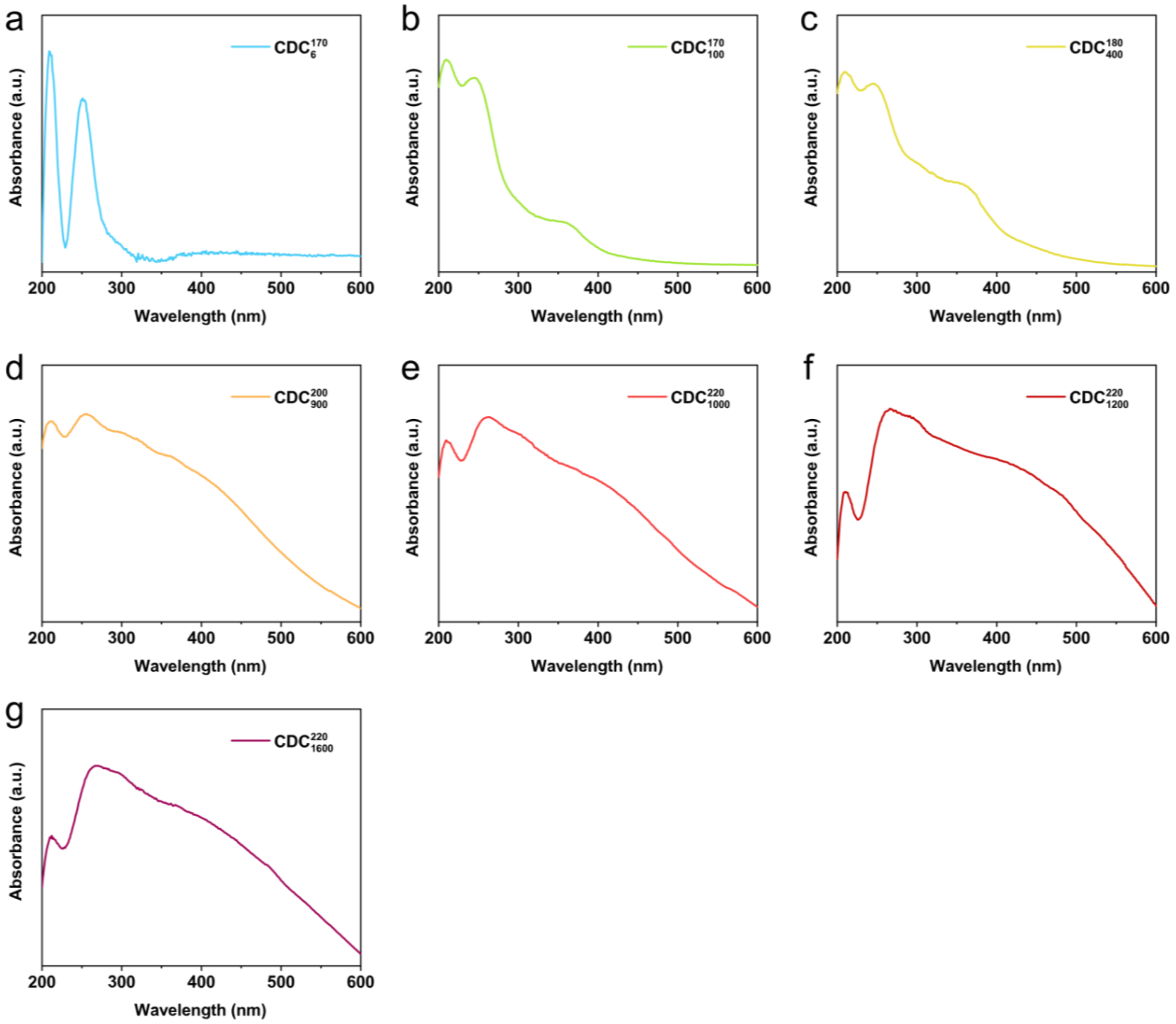

Figure S5. Normalized UV-vis absorption of a) CDC_{6}^{170}, b) CDC_{100}^{170}, c) CDC_{400}^{180}, d) CDC_{900}^{200}, e) CDC_{1000}^{220}, f) CDC_{1200}^{220}, and g) CDC_{1600}^{220} under ambient conditions.

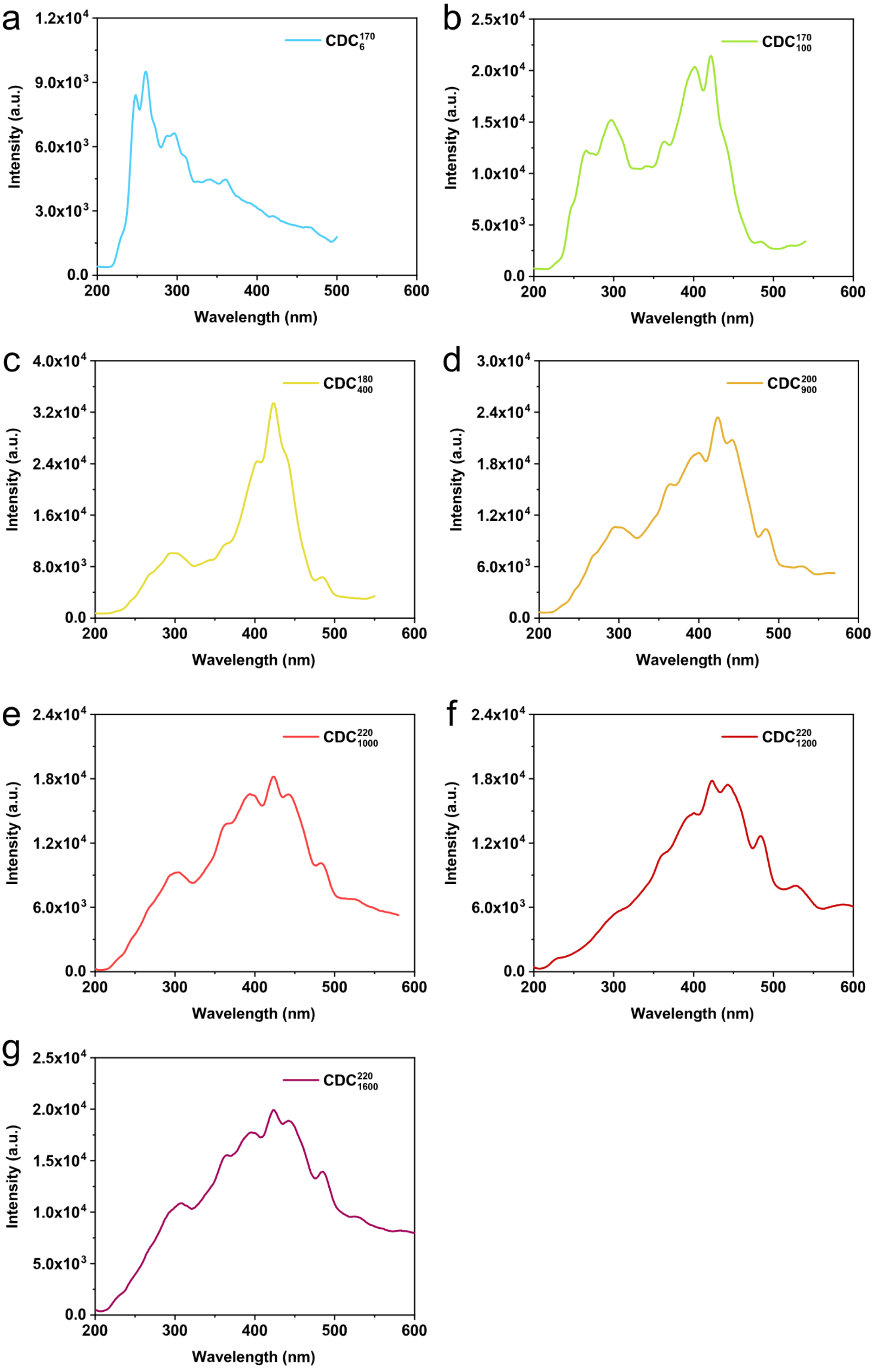

Figure S6. Phosphorescence excitation spectra of a) CDC^{170}_{6}, b) CDC^{170}_{100}, c) CDC^{180}_{400}, d) CDC^{200}_{900}, e) CDC^{220}_{1000}, f) CDC^{220}_{1200}, and g) CDC^{220}_{1600} monitored at 512, 564, 570, 592, 602, 621, and 638 nm, respectively, under ambient conditions.

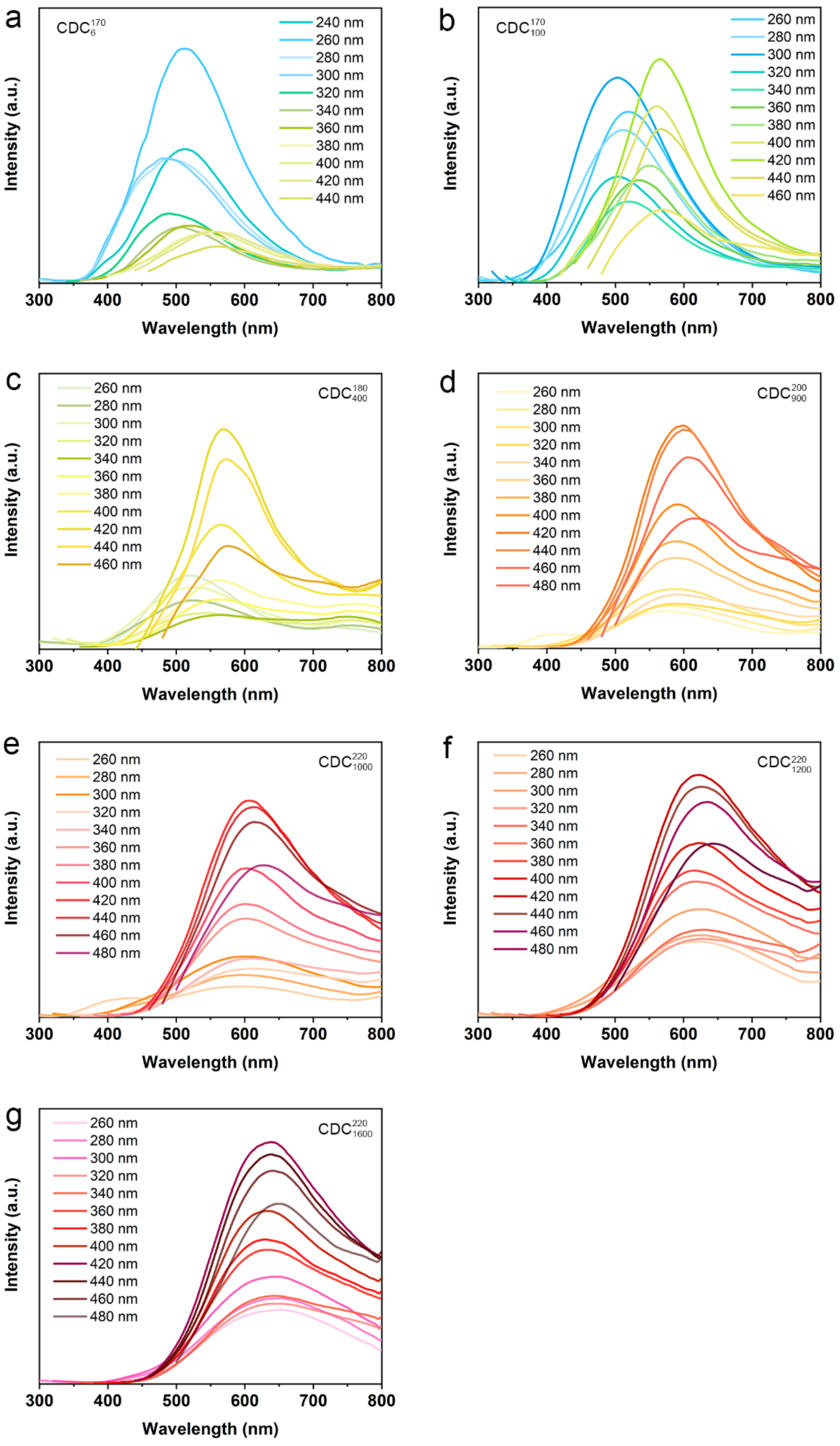

Figure S7. Phosphorescence spectra of a) CDC_{6}^{170}, b) CDC_{100}^{170}, c) CDC_{400}^{180}, d) CDC_{900}^{200}, e) CDC_{1000}^{220}, f) CDC_{1200}^{220}, and g) CDC_{1600}^{220} excited at different wavelengths under ambient conditions.

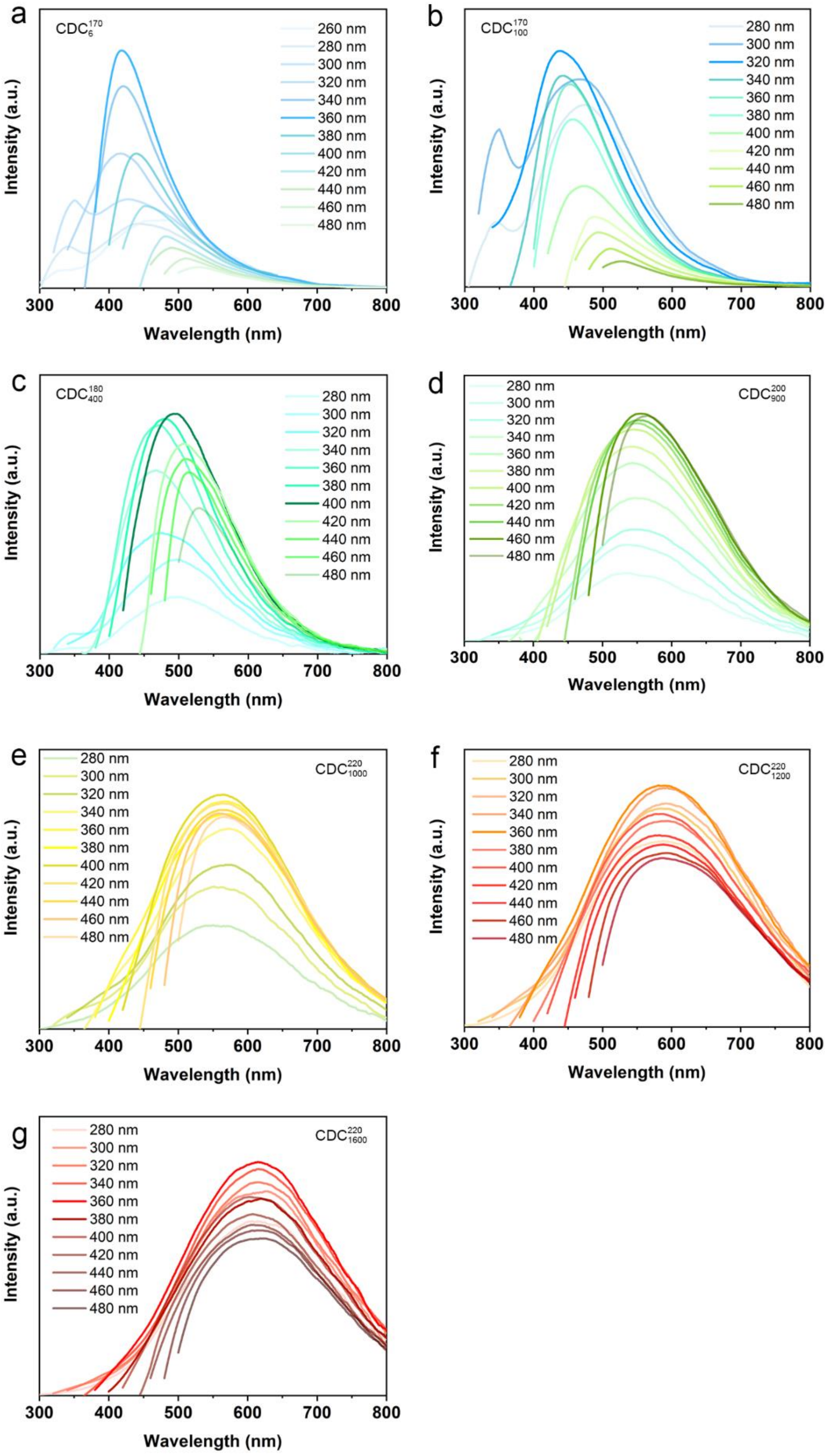

Figure S8. Steady-state photoluminescence spectra of a) CDC_{6}^{170}, b) CDC_{100}^{170}, c) CDC_{400}^{180}, d) CDC_{900}^{200}, e) CDC_{1000}^{220}, f) CDC_{1200}^{220}, and g) CDC_{1600}^{220} excited at different wavelengths under ambient conditions.

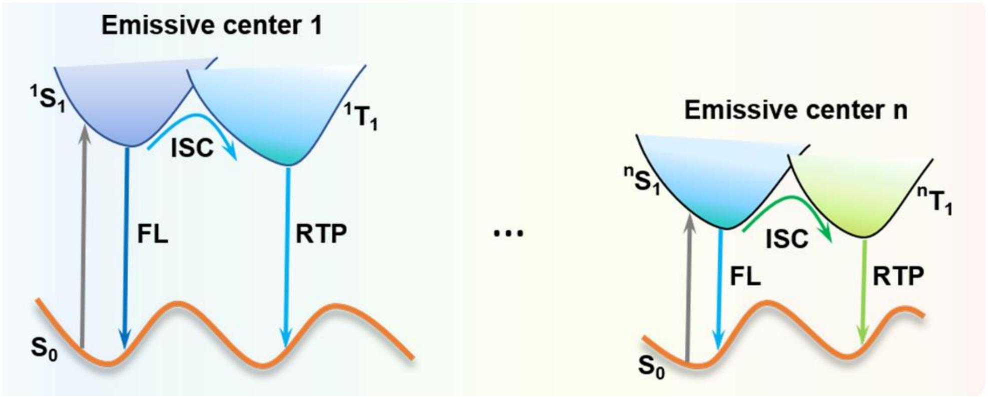

Figure S9. Proposed excitation-dependent RTP emission process of B-CD composites.

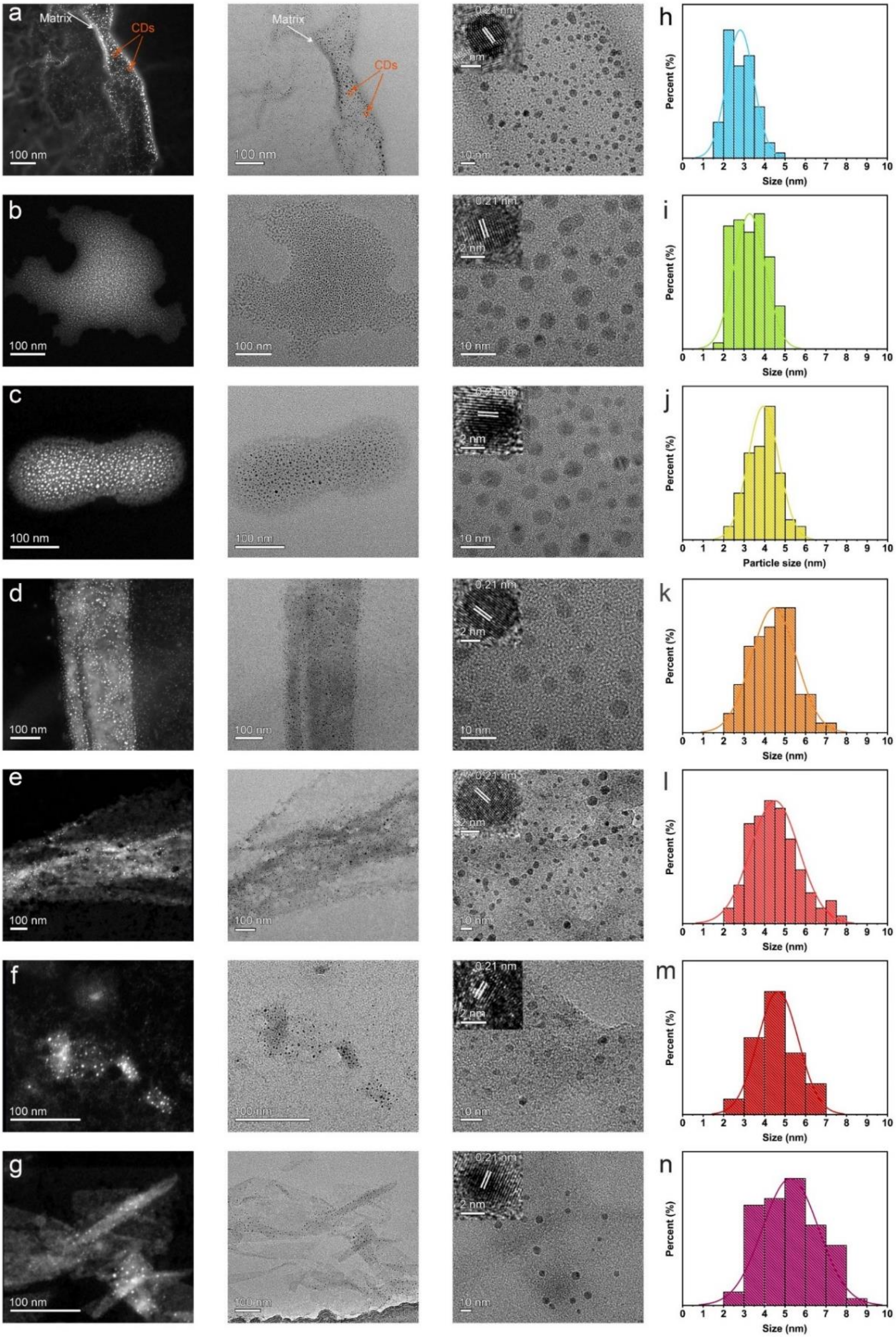

Figure S10. (a–g) HAADF-STEM, TEM, and HRTEM images (the inset images show the corresponding lattice fringes) of a) CDC_{6}^{170}, b) CDC_{100}^{170}, c) CDC_{400}^{180}, d) CDC_{900}^{200}, e) CDC_{1000}^{220}, f) CDC_{1200}^{220}, and g) CDC_{1600}^{220}. (h–n) Corresponding size distribution histogram.

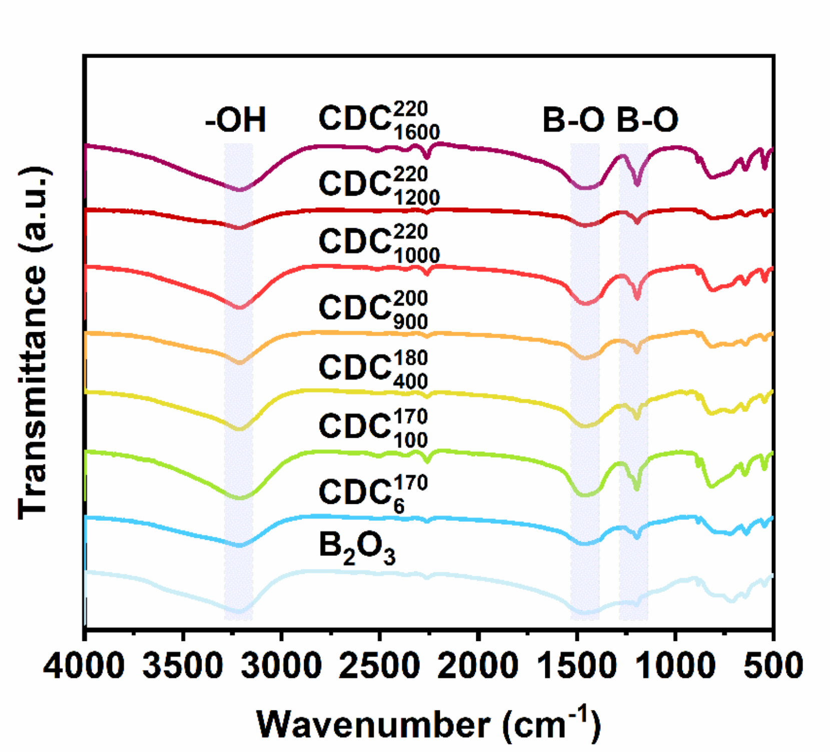

Figure S11. FTIR spectra of B_2O_3, CDC$^{170}_{6}$, CDC$^{170}_{100}$, CDC$^{180}_{400}$, CDC$^{200}_{900}$, CDC$^{220}_{1000}$, CDC$^{220}_{1200}$, and CDC$^{220}_{1600}$.

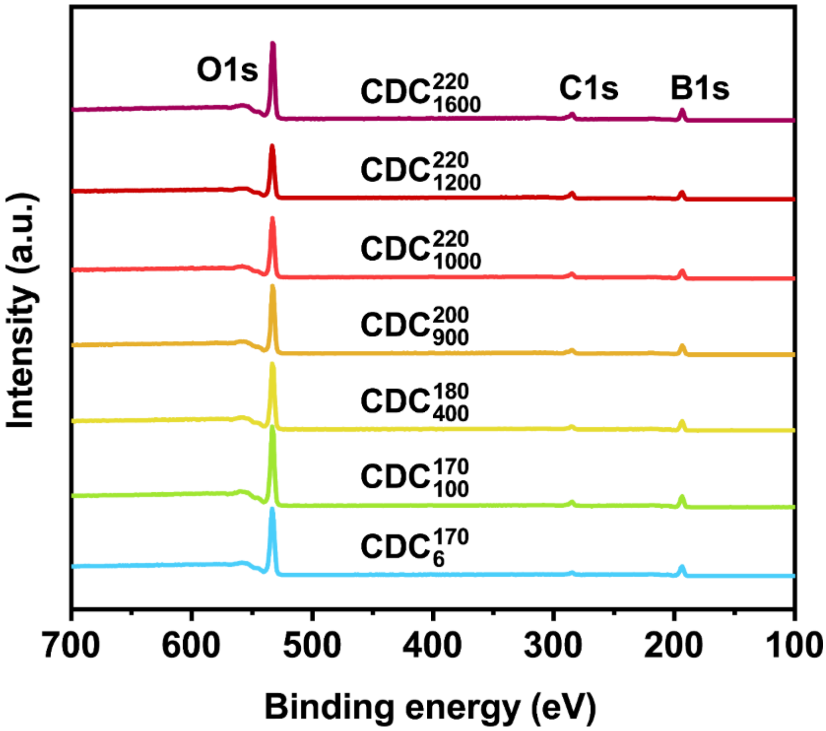

Figure S12. XPS survey spectra of CDC^{170}_{6}, CDC^{170}_{100}, CDC^{180}_{400}, CDC^{200}_{900}, CDC^{220}_{1000}, CDC^{220}_{1200}, and CDC^{220}_{1600}.

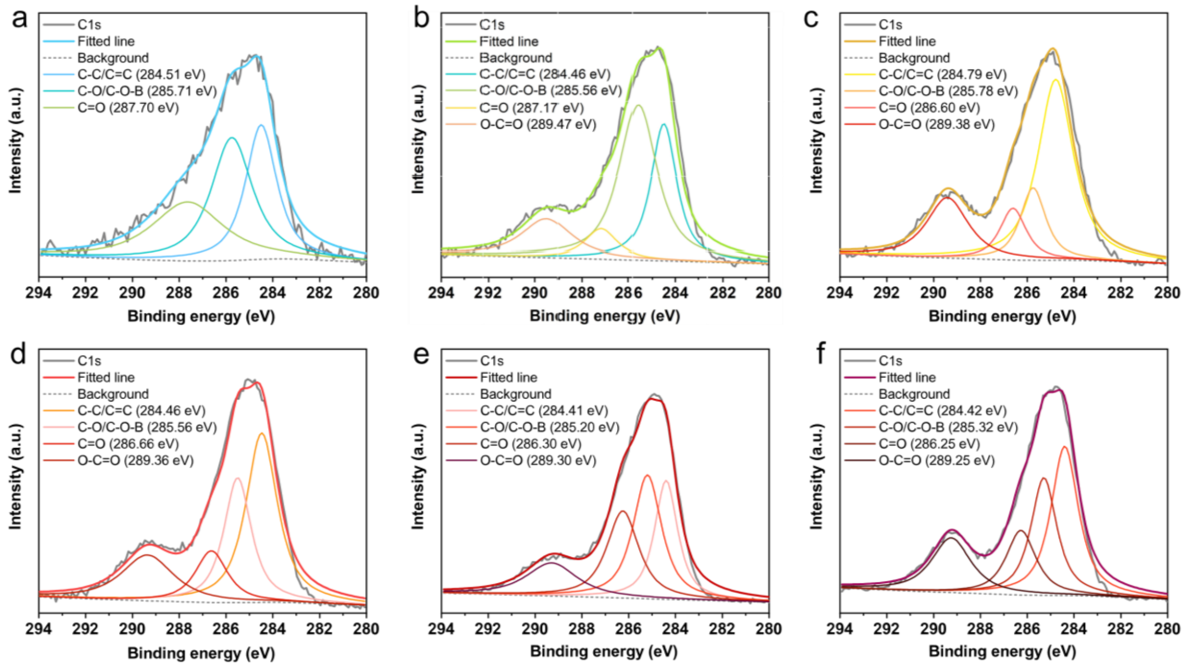

Figure S13. High-resolution XPS C1s spectra of a) CDC_{6}^{170}, b) CDC_{100}^{170}, c) CDC_{900}^{200}, d) CDC_{1000}^{220}, e) CDC_{1200}^{220}, and f) CDC_{1600}^{220}.

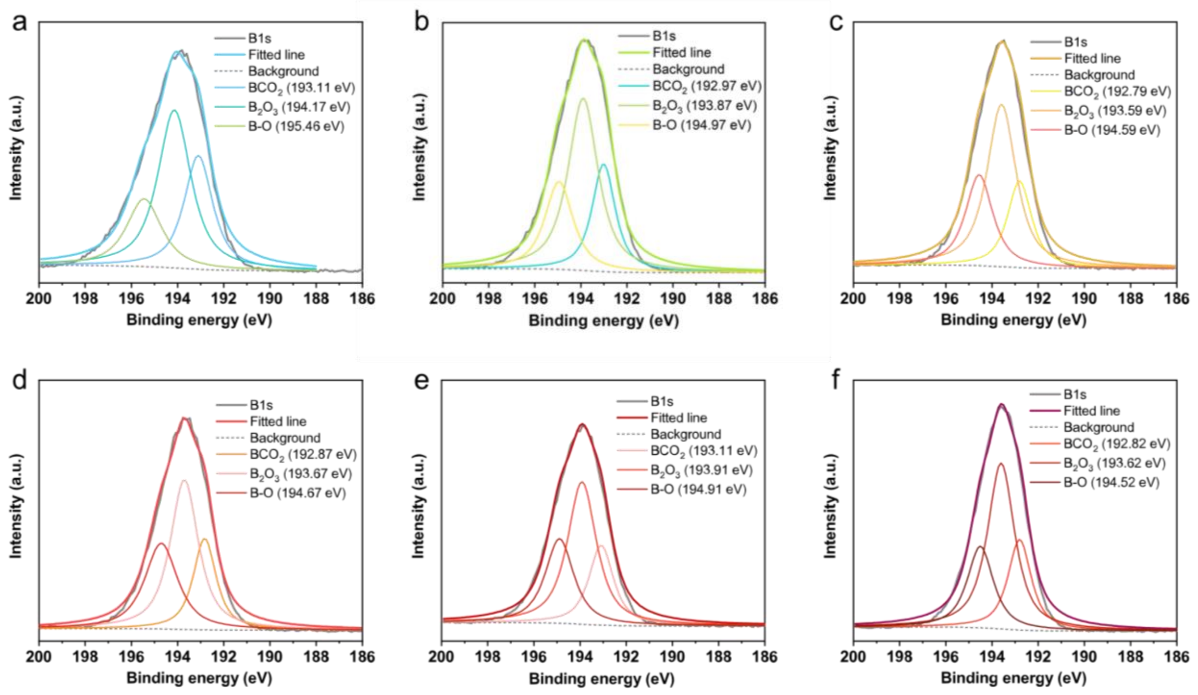

Figure S14. High-resolution XPS B1s spectra of a) CDC_{6}^{170}, b) CDC_{100}^{170}, c) CDC_{900}^{200}, d) CDC_{1000}^{220}, e) CDC_{1200}^{220}, and f) CDC_{1600}^{220}.

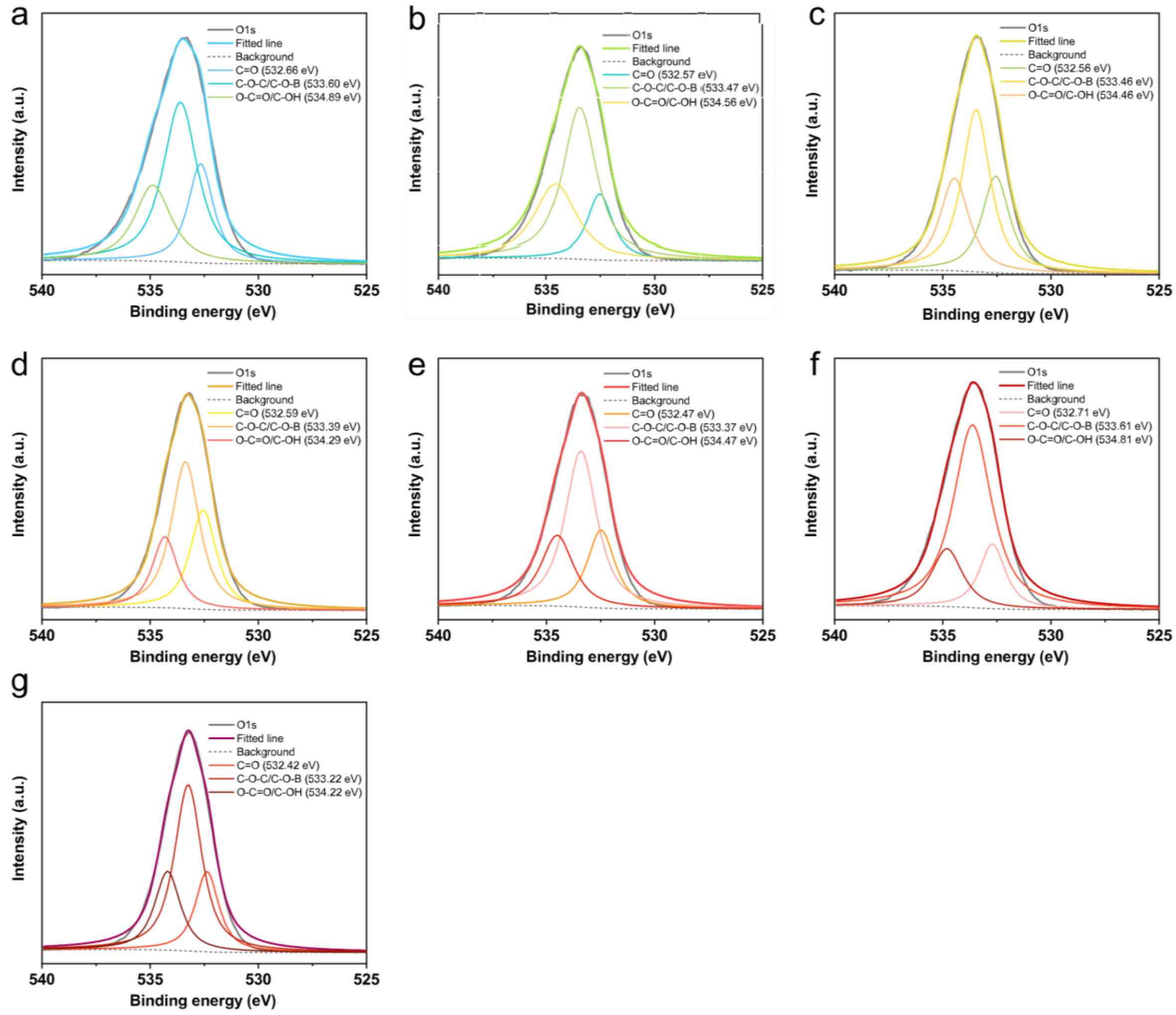

Figure S15. High-resolution XPS O1s spectra of a) CDC_{6}^{170}, b) CDC_{100}^{170}, c) CDC_{400}^{180}, d) CDC_{900}^{200}, e) CDC_{1000}^{220}, f) CDC_{1200}^{220}, and g) CDC_{1600}^{220}.

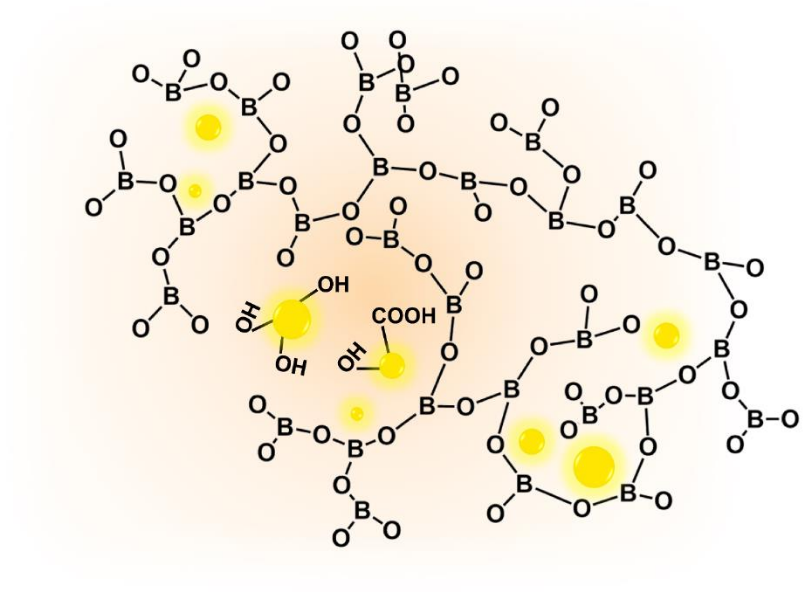

Figure S16. Proposed structure of B-CD composites.

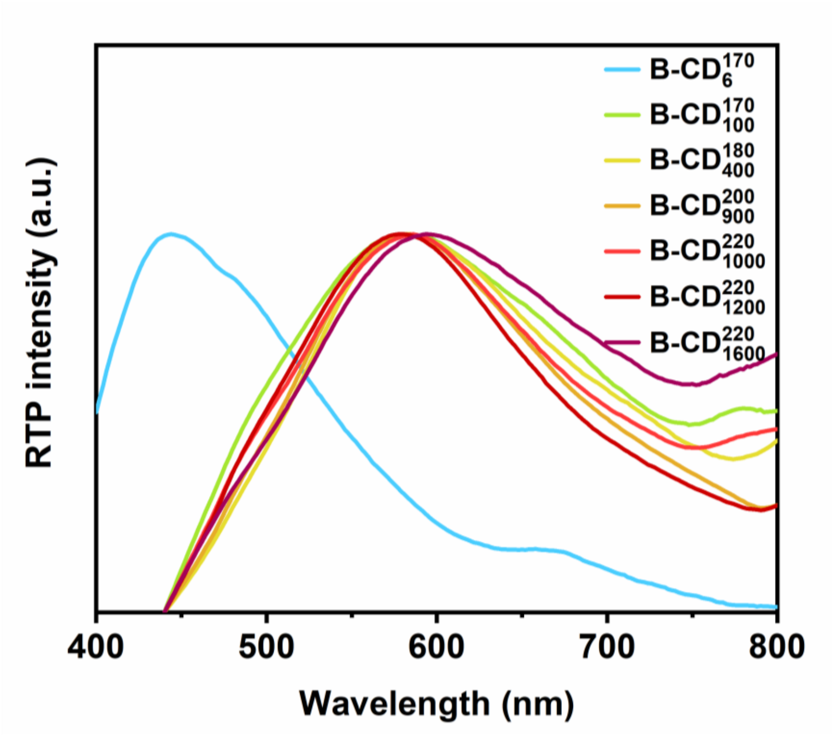

Figure S17. Phosphorescence spectra of B-CD composites prepared by melting the mixture of pure B-CDs and BA under ambient conditions.

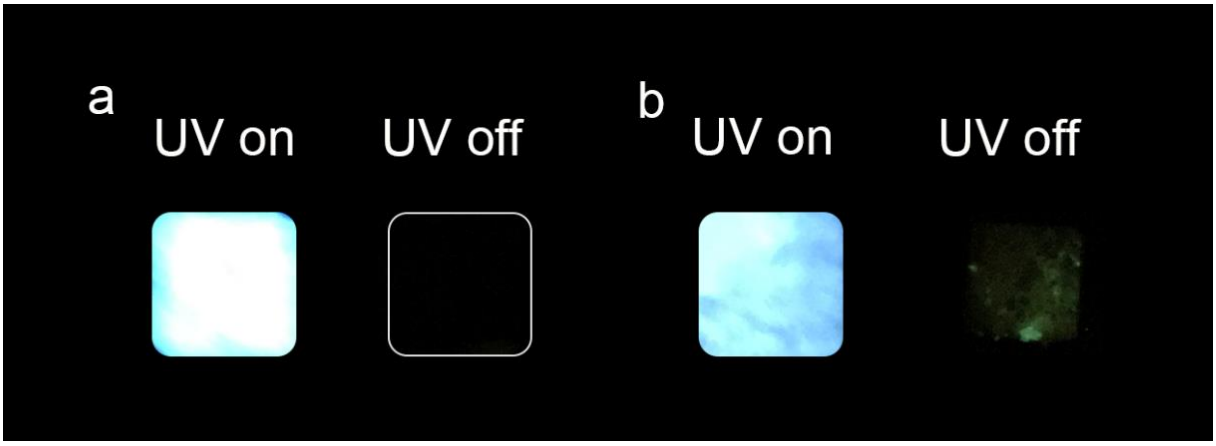

Figure S18. Fluorescence and phosphorescence images of compounds obtained by heating the mixture of CA (400 mg) and BA (6000 mg) at a) 100 and b) 150 °C under ambient conditions.

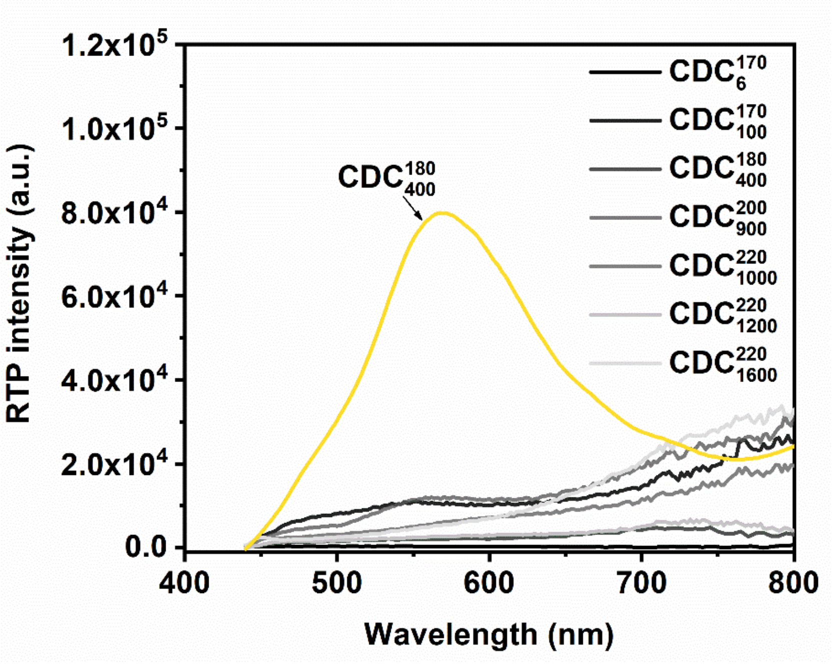

Figure S19. Phosphorescence spectra of CDC$_{6}^{170}$, CDC$_{100}^{170}$, CDC$_{400}^{180}$, CDC$_{900}^{200}$, CDC$_{1000}^{220}$, CDC$_{1200}^{220}$, and CDC$_{1600}^{220}$ after dispersed in water and freeze-dried under ambient conditions (yellow line represents the phosphorescence spectrum of as-made CDC$_{400}^{180}$).

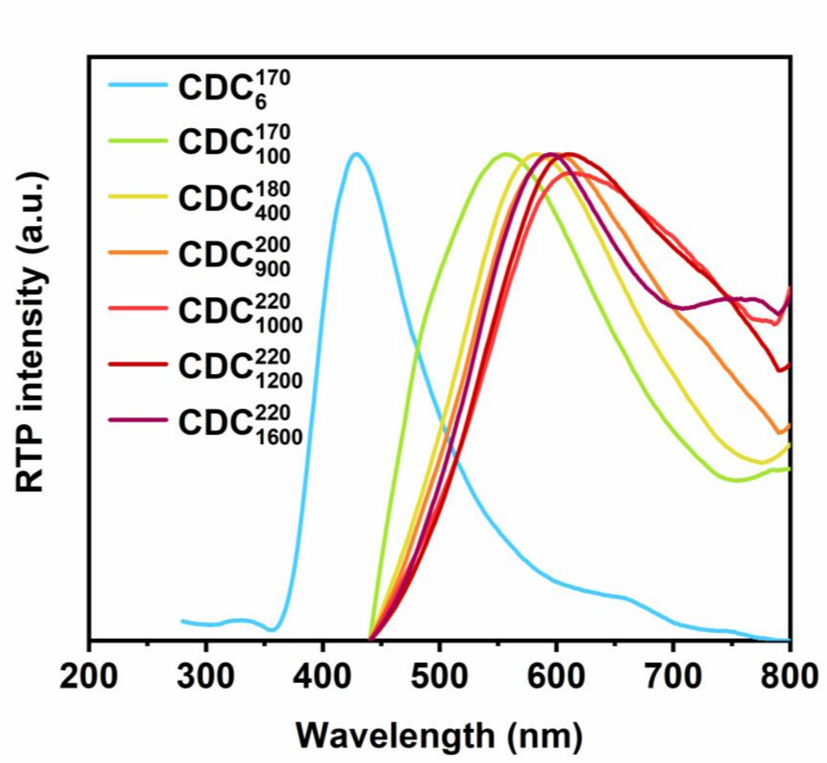

Figure S20. Phosphorescence spectra of CDC_{6}^{170}, CDC_{100}^{170}, CDC_{400}^{180}, CDC_{900}^{200}, CDC_{1000}^{220}, CDC_{1200}^{220}, and CDC_{1600}^{220} after freeze-dried and melted again under ambient conditions.

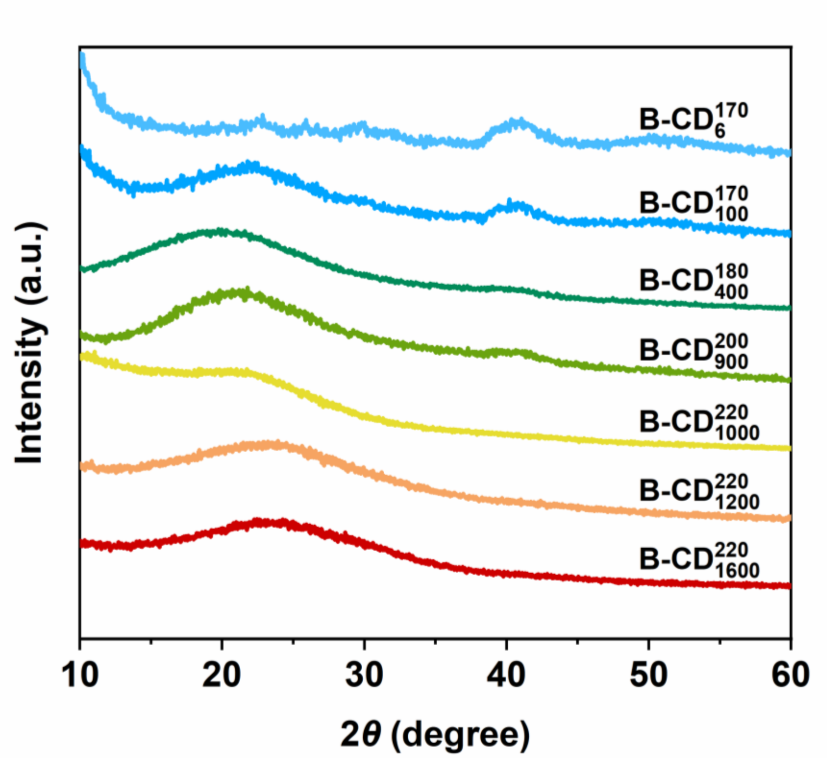

Figure S21. XRD patterns of B-CD$^{170}_{6}$, B-CD$^{170}_{100}$, B-CD$^{180}_{400}$, B-CD$^{200}_{900}$, B-CD$^{220}_{1000}$, B-CD$^{220}_{1200}$, and B-CD$^{220}_{1600}$ purified from the B-CD composites.

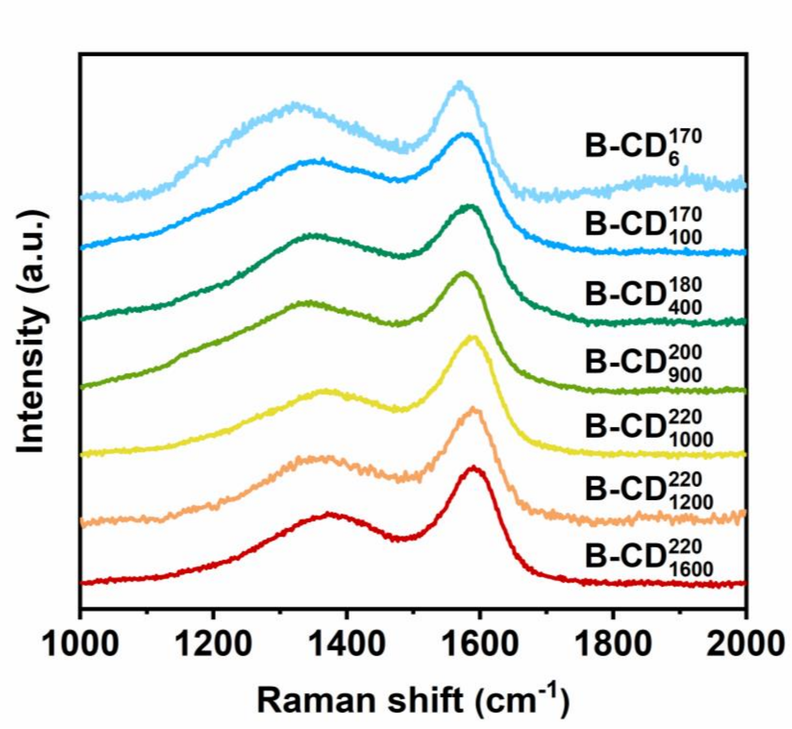

Figure S22. Raman spectra of B-CD$_{6}^{170}$, B-CD$_{100}^{170}$, B-CD$_{400}^{180}$, B-CD$_{900}^{200}$, B-CD$_{1000}^{220}$, B-CD$_{1200}^{220}$, and B-CD$_{1600}^{220}$ purified from the B-CD composites.

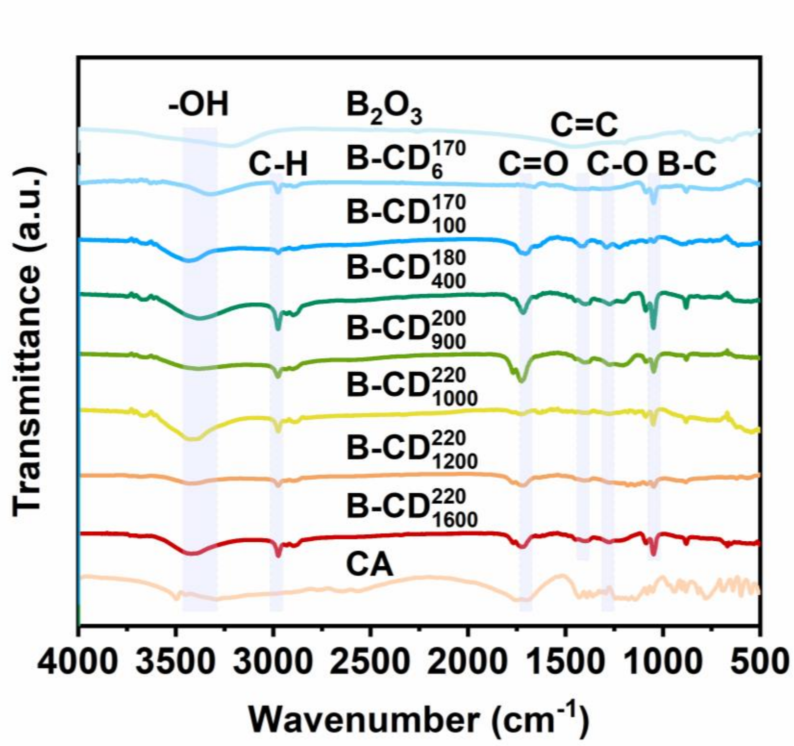

Figure S23. FTIR spectra of CA, B_2O_3, B-CD$_6^{170}$, B-CD$_{100}^{170}$, B-CD$_{400}^{180}$, B-CD$_{900}^{200}$, B-CD$_{1000}^{220}$, B-CD$_{1200}^{220}$, and B-CD$_{1600}^{220}$ purified from the B-CD composites.

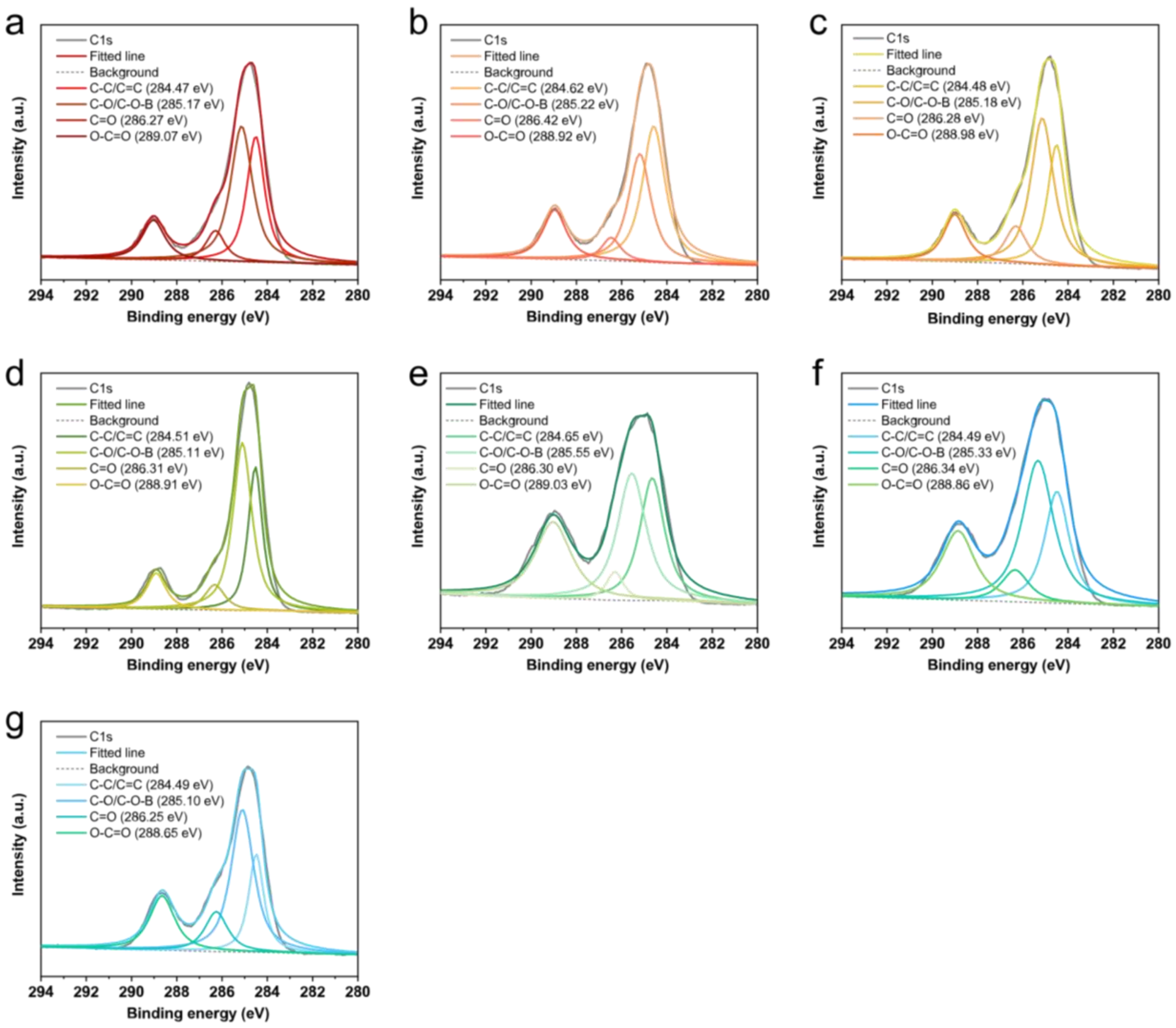

Figure S24. High-resolution XPS C1s spectra of a) B-CD$^{220}_{1600}$, b) B-CD$^{220}_{1200}$, c) B-CD$^{220}_{1000}$, d) B-CD$^{200}_{900}$, e) B-CD$^{180}_{400}$, f) B-CD$^{170}_{100}$, and g) B-CD$^{170}_{6}$ purified from the B-CD composites.

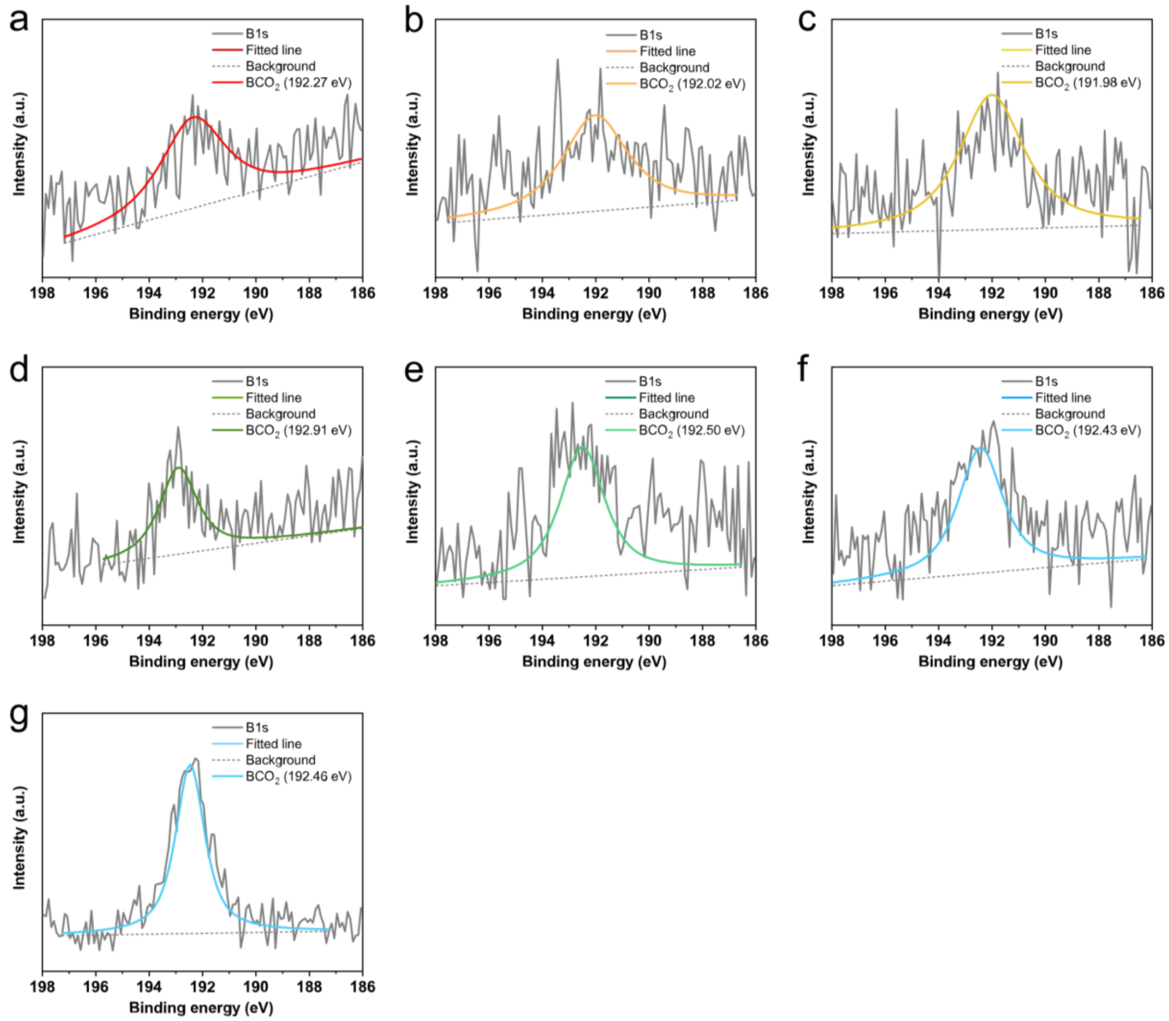

Figure S25. High-resolution XPS B1s spectra of a) B-CD$^{220}_{1600}$, b) B-CD$^{220}_{1200}$, c) B-CD$^{220}_{1000}$, d) B-CD$^{200}_{900}$, e) B-CD$^{180}_{400}$, f) B-CD$^{170}_{100}$, and g) B-CD$^{170}_{6}$ purified from the B-CD composites.

Table S12. XPS data analyses of the O1s spectra of B-CDs purified from the B-CD composites.

Sample	$B\text{-}CD_{6}^{170}$	$B\text{-}CD_{100}^{170}$	$B\text{-}CD_{400}^{180}$	$B\text{-}CD_{900}^{200}$	$B\text{-}CD_{1000}^{220}$	$B\text{-}CD_{1200}^{220}$	$B\text{-}CD_{1600}^{220}$
C=O (%)	28.16	32.29	36.85	48.61	50.06	54.63	58.16
C-O (%)	71.84	67.71	63.15	51.39	49.94	45.37	41.84

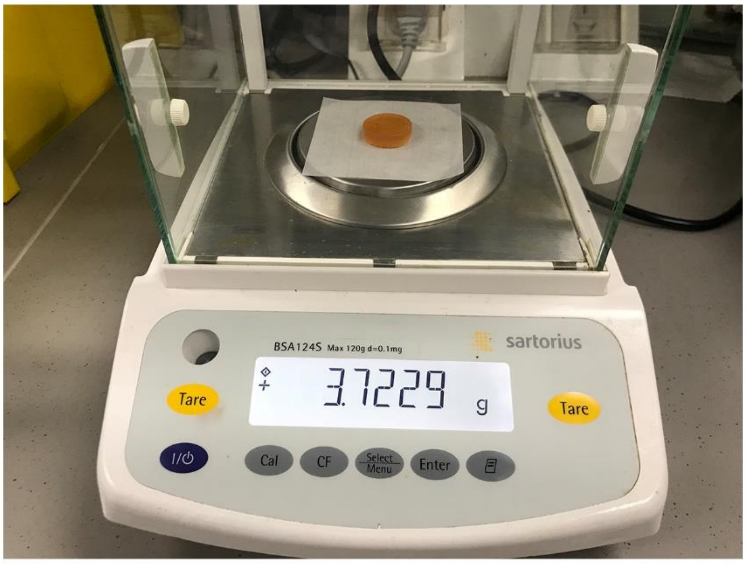

Figure S26. Gram-scale fabrication of CDC_{400}^{180}. 0.40 g of CA and 6.00 g of BA were mixed in 25 mL water and the mixture was heated at 180 °C for 5 h. This finally gave rise to a monolith with weight of 3.72 g.

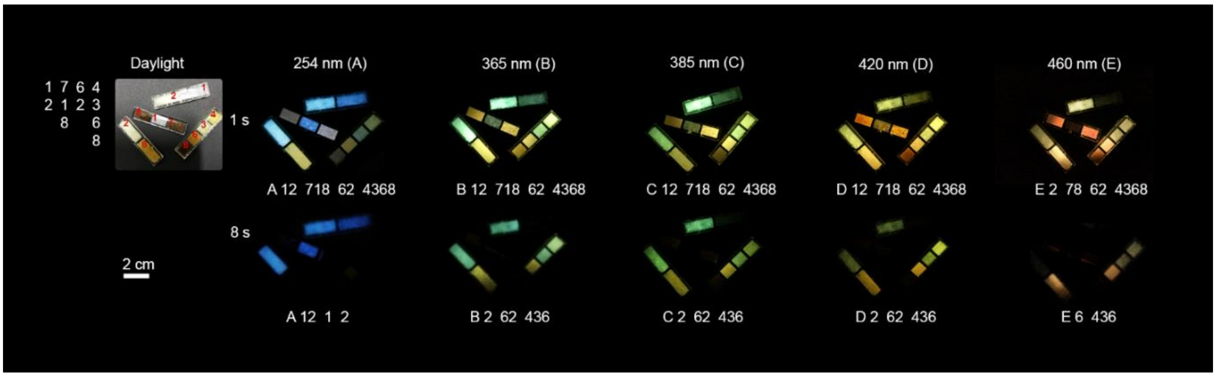

Figure S27. Randomly arranged barcodes derived from the B-CD composites for information encoding.

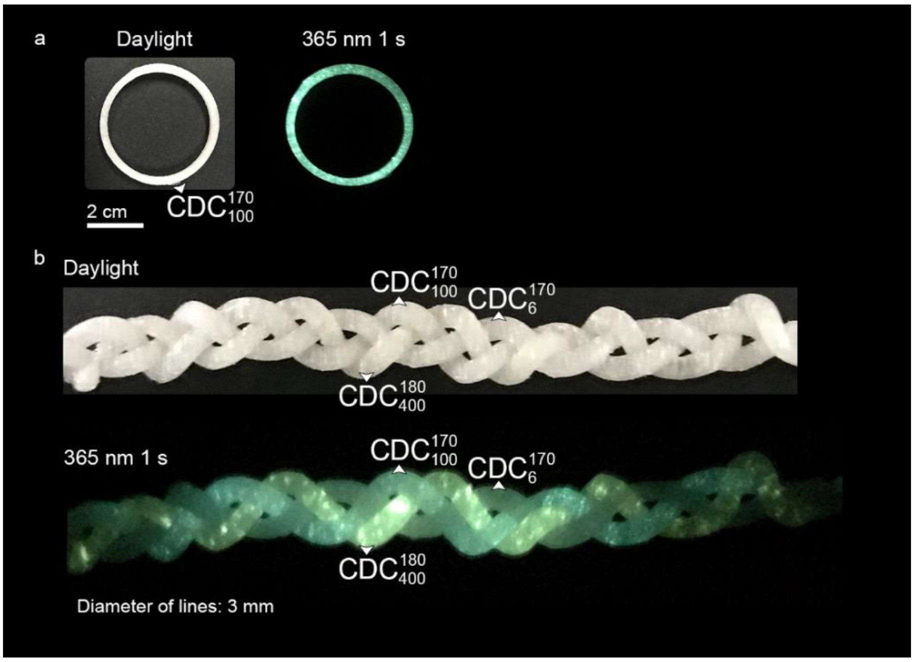

Figure S28. Ring and braided fabric fabricated by a gelatinous mixture of B-CD composites and poly(dimethylsiloxane).

[1] W. Ye, H. Ma, H. Shi, H. Wang, A. Lv, L. Bian, M. Zhang, C. Ma, K. Ling, M. Gu, Y. Mao, X. Yao, C. Gao, K. Shen, W. Jia, J. Zhi, S. Cai, Z. Song, J. Li, Y. Zhang, S. Lu, K. Liu, C. Dong, Q. Wang, Y. Zhou, W. Yao, Y. Zhang, H. Zhang, Z. Zhang, X. Hang, Z. An, X. Liu, W. Huang, *Nat. Mater.* **2021**, 1.
